# Cued to Act on Impulse: More Impulsive Choice and Risky Decision Making by Women Susceptible to Overeating after Exposure to Food Stimuli

**DOI:** 10.1371/journal.pone.0137626

**Published:** 2015-09-17

**Authors:** Martin R. Yeomans, Aaron Brace

**Affiliations:** School of Psychology, University of Sussex, Brighton, BN1 9QH, United Kingdom; Erasmus University Rotterdam, NETHERLANDS

## Abstract

There is increasing evidence that individual differences in tendency to overeat relate to impulsivity, possibly by increasing reactivity to food-related cues in the environment. This study tested whether acute exposure to food cues enhanced impulsive and risky responses in women classified on tendency to overeat, indexed by scores on the three factor eating questionnaire disinhibition (TFEQ-D), restraint (TFEQ-R) and hunger scales. Ninety six healthy women completed two measures of impulsive responding (delayed discounting, DDT and a Go No-Go, GNG, task) and a measure of risky decision making (the balloon analogue risk task, BART) as well as questionnaire measures of impulsive behaviour either after looking at a series of pictures of food or visually matched controls. Impulsivity (DDT) and risk-taking (BART) were both positively associated with TFEQ-D scores, but in both cases this effect was exacerbated by prior exposure to food cues. No effects of restraint were found. TFEQ-D scores were also related to more commission errors on the GNG, while restrained women were slower on the GNG, but neither effect was modified by cue exposure. Overall these data suggest that exposure to food cues act to enhance general impulsive responding in women at risk of overeating and tentatively suggest an important interaction between tendency for impulsive decision making and food cues that may help explain a key underlying risk factor for overeating.

## Introduction

There has been a dramatic increase in the incidence of overweight and obesity worldwide [1,2], which can be attributed to an imbalance between energy intake and expenditure. Although the causes of this imbalance are complex, it is widely accepted that short-term over-eating is a key factor [3,4]. However, there is clear variation in susceptibility to weight gain [5,6]. Identification of the phenotypic characteristics of those who are both prone and resistant to overeating has been recognised as critical to future development of targeted approaches to both prevent future weight gain and in aiding the development of improved methods for promoting weight loss [5].

One area that has been the focus of considerable research interest is impulsivity, since differences in the ability to control behaviour could plausibly relate to poor dietary control. However, the current literature on the relationship between impulsivity and eating behaviour is complex. Measures of both trait impulsiveness (i.e. self-reported longer term impulsive behaviour) and impulsive responding (impulsive behaviour in acute behavioural tests) have been found in some studies to be positively associated with body-size [7–11], yet others fail to find this relationship. Moreover, even where some evidence for an association with obesity was reported, this was not reflected in all measures of impulsivity. For example, in a contrast on measures of impulsivity between obese and normal weight women, a difference in response inhibition was only found in the final session of four tests on a Stop-signal task, with no difference between groups on three measures of self-reported impulsiveness [7]. Likewise, higher scores on various measures of impulsivity have been associated with binge eating [12,13], bulimia nervosa [14–16], measures of food reinforcement [17,18] and scores on questionnaire measures of uncontrolled eating [19,20] in some studies, but not others [21–23].

Impulsivity is multi-faceted [24,25], and a number of behavioural tasks have been developed to capture different facets of impulsive response. The delay discounting task (DDT), where impulsive individuals choose an immediate smaller reward over a delayed larger reward [26], measures impulsive choice and is often interpreted as evidence of sensitivity to immediate reward. Performance on the DDT has been related to overeating or weight in some studies: obese women, but not men, showed greater delay discounting [27] and women scoring higher on a self-report measure of uncontrolled eating (the disinhibition scale of the Three Factor Eating Questionnaire: TFEQ-D [28]) were also more impulsive on the DDT [20]. DDT performance also has some predictive value: impulsive choice on the DDT predicted lack of response in weight loss programmes for overweight boys [18]. However, not all studies measuring DDT performance find significant group differences either with obese participants [7] or as a function of TFEQ-D score [19]. Even studies that have reported differences in DDT performance between groups can be hard to interpret: for example, both obese binge-eating and non binge-eating were more impulsive than normal weight controls on the DDT, but this effect was not significant once educational status was controlled for [13].

Another key component of impulsivity reflects the failure to inhibit an inappropriate response to prepotent stimuli (impulsive action), usually indexed by performance on Go/No-Go (GNG) or Stop signal tasks [29]. Again, there is some evidence that impulsive action may be related to overeating. For example, as discussed earlier, obese women scored more impulsively on a Stop-signal task than did normal-weight women [7] and BMI correlated with poor performance by adolescent girls on a food-related GNG task [9]. Stop-signal and GNG performance have also been found to correlate with acute food intake [30]. Greater impulsive responding on the GNG has also been associated with binge-eating in girls and sweetened beverage consumption in boys [31]. However, other studies have failed to find evidence that poor inhibitory control measured using GNG or Stop-signal tasks relates to disordered eating: patients with bulimia, binge eating disorder or obesity did not show weaker inhibitory control compared with controls [21–23]. And whereas women scoring high on TFEQ-D were more impulsive on the DDT, they were not impulsive on a Stop-signal task [19]. Thus, as with DDT, the relationship between impulsive action and eating is complex and contradictory.

What then might explain these contradictory findings? One possibility is that impulsive choice and inhibitory control may be acutely sensitive to reward-related cues in the environment, and that studies differ in the degree to which participants are exposed to reward-related cues within the test session. Although no previous study was designed specifically to test this hypothesis, several studies have included measures that are relevant to this idea. Most relevant are studies that have manipulated appetitive state at the time of testing. In two related studies, it was firstly shown that participants who were more impulsive on a Stop-signal task ate more at an intake test but only when in a food-deprived rather than sated condition, and secondly those classified as impulsive on the Stop-signal task purchased more energy from a virtual supermarket only when hungry [32]. Crucially for the present study, this interaction between hunger state and impulsivity may be exacerbated by food cues: hungry participants made more commission errors in a GNG task when food rather than control images served as distractors [33]. There is also evidence that food-cue reactivity and impulsivity are related. Thus food intake in a bogus taste test was only associated with a measure of food cue reactivity in people scoring impulsively on the DDT [34]. These studies all imply that the impact of impulsivity depends on either acute appetitive state or the presence of food cues. However, in all of these studies impulsivity was treated as a trait variable and was typically used as a potential moderator of the relationship between the manipulation of appetite and/or presence of food cues and food intake in a test. This leaves open the key question of whether impulsive responding itself is modified by the presence of food cues. One study which attempted to address this [35] had healthy women, classified as restrained or unrestrained eaters, complete the Stop signal task in four blocks with a short break between blocks. All participants then viewed a selection of snack foods for 2 minutes between blocks 2 and 3, with no significant effects on Stop signal performance, which was stable across blocks 2–4. However, the study focussed on restrained eating, which our recent work finds does not relate well to impulsivity [19,20], and only measured inhibitory responding. Moreover, all participants were exposed to food cues, and so the study relied on group differences in response to food rather than testing effects of food exposure per se.

The present study was an attempt to explore the effects of acute exposure to food, relative to matched non-food, cues on subsequent measures of impulsive choice (DDT) and impulsive action (GNG). If impulsivity reflects a trait over-sensitivity to reward and inability to inhibit responding, we predicted that women prone to uncontrolled eating (here indexed by higher scores on the TFEQ-D subscale) would be more impulsive than would women scoring lower on TFEQ-D regardless of cue-exposure. If impulsive behaviour is exacerbated by expectation of reward triggered by the sight of food-related cues, then we predicted that participants would act more impulsively after exposure to food than control cues. However, we also recognised that food cue exposure might have effects on behaviour beyond enhanced impulsivity. In particular, cue exposure could lead to enhanced risky decision making independent of the specific behavioural measures of impulsivity. We therefore also included a behavioural measure which has been interpreted as risky decision-making, the Balloon Analogue Risk Task (BART [36]). The BART task is seen as a measure of individual differences in propensity to make risky decisions, and high BART scores have been found to relate to risk-taking in adolescence [37], smoking [38] and use of abused drugs [39]. Although previously we found no overall difference in BART performance between women scoring high or low on TFEQ-D [19], in that study women who had to control their eating prior to the test showed greater risk-taking, which suggests exposure to food cues might lead to riskier behaviour on the BART. Finally, we included a questionnaire measure of reward reactivity and inhibition (the Behavioural Inhibition System—Behavioural Approach System—BIS/BAS, [40]) to assess both individual differences on that measure in relation to the behavioural tests and assess whether responses to this trait measure might also be affected by exposure to food cues.

## Methods

### Design

Responses on two behavioural measures of impulsive responding (DDT and GNG), a self-report measure of impulsiveness (BIS-BAS questionnaire) and a measure of risky decision making (the BART) were recorded after exposure to a series of pictures of food (condition Food) or matched neutral pictures (condition Control) by women classified depending on their scores on both the disinhibition and restraint scales of the TFEQ.

### Participants

Participants were 96 women volunteers, mostly students or staff at University of Sussex who had previously expressed an interest in participating in studies in Psychology but who has not previously completed studies involving measures of impulsivity. The sample size was based on a power analysis of the data from our original finding that TFEQ-D and DDT performance were correlated [20]. Potential participants had completed the TFEQ as part of an on-line recruitment questionnaire within the previous 6 months, and were sent an email asking if they would be interested in taking part in a short study on mood and cognitive performance, and the participants were the first 96 to respond positively. Forty-eight of these respondents were assigned at random to either the Food or Control conditions. All participants gave their written informed consent prior to testing and were entered into a prize draw for a cash reward as a thank you for their participation. The procedure was approved by the University of Sussex Ethics committee (CREC), and the study conducted according to the principles in the Declaration of Helsinki.

### Measures of impulsive responding

#### Go-NoGo task

In the GNG task, participants were presented with a series of visual stimuli (blue ovals) in one of two orientations. On Go trials, the principal axes of the oval was vertical, and on these trials participants were instructed to respond (by pressing the key-board space bar) as quickly as possible on stimulus presentation. On No-go trials, the same stimulus was presented but with the main axes at 45° and here participants were required not to respond. The stimuli were presented in five blocks: the initial block comprised of 12 Go and 12 NoGo presentations in random order. To increase the likelihood that responding on Go trials became habitual, and so increasing the chance of errors on NoGo trials, Block 2 comprised 24 Go stimuli. The third block was again a mixture of 12 Go and 12 No-go trials, followed by a second block of 24 Go only trials and a final mixed block of 12 Go/12 NoGo trials. On each trial, the stimuli were displayed until the participant responded, with a 2000ms timeout, and each presentation was followed by an inter-stimulus interval of 1000ms. The task generated three measures of interest: commission errors, where participants incorrectly responded on NoGo trials, omission errors where participants failed to respond on Go trials and response rate defined as the time taken to respond on Go trials. The task was programmed using E Prime 1.2 (Psychology Software) running on a Windows PC using Windows XP.

#### Delay Discounting Task (DDT)

The DDT used in this study was developed from that used in previous studies at Sussex (Yeomans et al., 2008), and used a series of choices between a fixed £100 reward at six different delays (1, 7, 30, 90, 180 and 365 days) and an immediate variable amount of money (£0.10, £2.50, £5.00, £10.00, £15.00, £20.00, £25.00, £30.00, £35.00, £40.00, £45.00, £50.00, £55.00, £60.00, £65.00, £70.00, £75.00, £80.00, £85.00, £90.00, £95.00, £100.00) to determine an indifference point for each delay, defined as the amount of money which fell between a clear preference for the delayed and immediate rewards. Choices were presented on a computer touch-screen as two coloured boxes, with the immediate sum on the left and the fixed £100 and associated delay on the right, and participants indicated which of these items they preferred by touching their preferred choice. The quantity of money on offer was increased from previous versions following concerns that the small sums used previously (£10 delayed choice) might have limited the ability to detect impulsive choice. The task was programmed using E-Prime 1.2 (Psychology Software) run on a Windows PC using Windows.

Indifference points at each delay were estimated as the breakpoint between the lowest value where the delayed reward was consistently preferred and the highest value where the immediate reward was preferred. Thus if at 30 days delay all values of £50 or more were preferred to the delayed £100 reward but the £100 delayed reward was preferred to an immediate reward of £40 or less, the indifference point was estimated as £45.00. On the rare occasions where choices were inconsistent, the breakpoint was determined as the average of the sums between a clear preference for the delayed reward (defined as at least three sequential preferences for the £100) and a similarly clear preference for the immediate reward (i.e. three sequential choices for the smaller reward). The area under the curve (AUC) method was used to convert indifference points into a single measure of impulsive choice as this approach is theoretically neutral (Myerson, 2001). Higher impulsive choice is expressed as a smaller AUC value.

#### BART

The version of the BART used in this study was developed and provided by Lejuez and colleagues [36]. The goal of the BART was to “earn” hypothetical money by pumping a balloon displayed on-screen. Every pump of the current balloon earned £0.05, and the participant had the option to “transfer” their accumulated money from that trial to their bank, ending that trial. However, pumping up the balloon entailed a risk that the balloon would burst, and all money gained on that trial lost, with the explosion point varying across trials at random. The procedure consisted of 30 balloons, with the potential to earn £60.00 in total. Risk-taking is operationalized in two ways: the first is by explosions incurred during the test, and the second by the Adjusted Average Pumps, which is the average number of pumps excluding balloons that have exploded. Due to computer problems, 19 participants did not complete this task.

### Self report measures of eating behaviour and impulsivity

#### The Three Factor Eating Questionnaire (TFEQ)

The original TFEQ [28] was used to define women depending on their tendency to restrict their intake in relation to weight concerns (TFEQ restraint scales), tendency to exhibit uncontrolled eating (TFEQ disinhibition scale) and sensitivity to hunger (TFEQ hunger scale). The TFEQ (also known as the Eating Inventory) is a widely used questionnaire measure of eating and dieting behaviours, and has been cited in over 2500 studies to date. The TFEQ combines 36 items with yes/no responses, 14 items with four option responses (which are scored as 0 or 1) and one item scored from 0–5 (again scored 0 or 1). Based on the original published structure there are 21 items for restraint, 16 for disinhibition and 14 for hunger, based on the consistency reported in the original development [28]. Some people have questioned the validity of the scale structure: Bond et al. [41] suggested that each sub-scale could be further subdivided, and an analysis in obese and overweight Swedish participants found significant overlap between items from the disinhibition and hunger scales [42]. However, the conflicting recommendations either that a shorter version be used or that the three factors are further subdivided to overcome these potential inconsistencies has not been widely accepted. To allow outcomes to be readily compared with the existing published literature, we therefore used the original TFEQ.

#### BIS-BAS

The BIS/BAS questionnaire [40] is a 24-item self-report measure that generates three measures of sensitivity to reward (BAS Drive, BAS Fun Seeking, and BAS Reward Responsiveness) and a single measure of sensitivity to punishment/avoidance (BIS). These scales have been used widely in clinical studies of impulse control but have been used more rarely in studies relating reward sensitivity to individual differences in eating. The derived structure of the BIS/BAS scales has adequate internal consistency [40].

### Cue-Exposure

**Fig 1:**
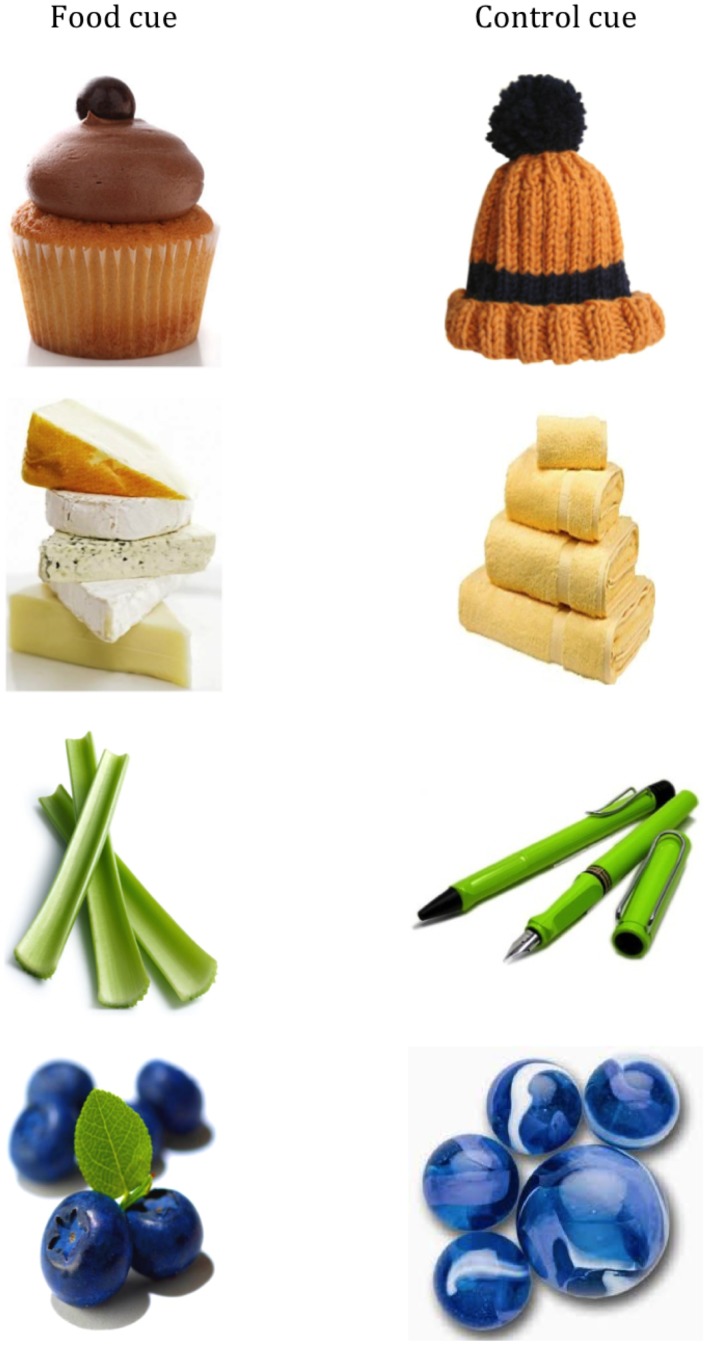
Four examples of the food items and matched controls used during the cue-exposure phase.

To expose participants to images of food or matched controls, a series of ten pictures of foods that would be considered unhealthy, and a further 10 images of healthy foods, were selected. We were also concerned that simply showing people a set of food images only might have lead to demand effects that could have influenced their subsequent behaviour: to reduce such demand effects all participants were also exposed to 10 neutral (i.e. non-food) images, which were interspersed with the food images. Each of the food images were matched on visual inspection with control images un-related to food but with similar visual features. The 20 food images used, and their controls, are listed in Table 1, and example images are shown in Fig 1. All images were adjusted to the same approximate size (9.5cm x 7cm), and were displayed in the centre of the screen on a white background using Microsoft Powerpoint with image resolution of 300dpi (the relevant Powerpoint files are included as S1 and S2 Files). Images were all copyright-free pictures from internet searches and each pair of stimuli (i.e. food and matched control) were visually similar in colour, complexity and visual appearance. Stimuli were presented in a pre-set order with healthy, unhealthy and control stimuli distributed in a semi-randomised order. We recognised that using a fully randomised order would have raised potential confounds since primacy and recency effects would have predicted greater impact from stimuli early and late in the presentation, and a truly random sequencing could have resulted in no food stimuli at those key times. Instead, firstly we divided the stimulus sequence into sets of three images (one from each category) and then randomised the order within each set of three. Secondly we randomly allocated one image from each category to each of these sets. The final sequence was then viewed by all participants. Each image was presented for 10 seconds. To ensure participants paid attention to these stimuli, they were informed that these stimuli would be part of a memory task later in the study. To make this plausible, they were later presented with a single A4 printed sheet with 60 images, the 30 they had been exposed to and 30 controls. For the Food-cue group, the control images were the equivalent non-food items, and vice versa for the Control group. The remaining 20 items were the 10 neutral items from exposure and a further 10 neutral items which were similar to the exposed neutral items.

**Table 1:** Cue stimuli used in the study.

Unhealthy food items		Healthy food items	
Food item	Control	Food item	Control
Muffin	Bobble hat	Orange	Tennis ball
Chocolate squares	Chess board	Dried apricots	Orange buttons
Pick-iced cake	Pink pouffe	Cabbage	Green balloon
Trifle	Vase of flowers	Blueberries	Blue marbles
Pile of cheese	Pile of towels	Broccoli spike	Shaving brush
Chocolate doughnut	Wooden napkin ring	Strawberry	Red purse
Tortilla crisps	Maple leaves	Celery sticks	Green pens
Swiss roll	Log	Banana	Washing-up glove
Jar of sweets	Candle jar	Rice cakes	Rolls of tape
Hamburger	Pile of laundry	Prunes	Lump of coal

### Procedure

All testing was conducted in small windowless test cubicles. Participants were invited to the laboratory at a pre-defined time and were seated in front of the computer. After reading an information sheet and signing consent, they completed a series of computerised mood ratings which had two purposes: a) to fit with the study cover story and b) to give a record of rated appetite at the onset which could be used to control for any spurious group differences in hunger prior to testing that could have confounded the cue-exposure procedure. Appetite ratings were how hungry, full and thirsty participants felt, along with ratings of how calm, happy, clearheaded, anxious, tired, energetic, lively and alert, all rated on 100pt computerised visual analogue scales end-anchored nota at all (0) and extremely (100), presented using Sussex Ingestion Pattern Monitor (SIPM 2.015, University of Sussex) experimenter software. The mood ratings were included to comply with the cover-story that the project examined how mood modified cognitive performance, and only the hunger rating is included in analysis as differences in appetitive state could have impacted responses to the food cues. Participants were then informed they would complete a memory test and would now be shown a series of pictures. They then viewed the 30 picture stimuli. Immediately afterwards they completed the GNG task followed by the DDT, BART (for those completing this) and the BIS/BAS questionnaire. To comply with the cover story they were given the picture identification sheet and had 2 minutes to identify as many pictures as they could from the initial memory set (data not analysed). Age, height and weight were recorded followed by debriefing.

### Data analysis

Complete DDT data were collected for all 96 participants, but computer failure meant one set of GNG data were lost, and one participant failed to complete all of the BIS/BAS questionnaire, while 75 participants completed the BART. Initial analysis of DDT data contrasted indifference scores as a function of delay between the two test conditions, including TFEQ-D and TFEQ-R scores as covariates using ANCOVA. The relationship between key measures of impulsive and risky responding (DDT AUC; GNG reaction time, commission and omission errors; BART explosions and adjusted average pumps; BIS/BAS scores) were assessed using step-wise linear regression with condition (Food or Control), TFEQ-D, TFEQ-R and TFEQ-H scores, the three cue*TFEQ interactions, rated hunger and interactions between hunger and TFEQ scores. Age, BMI at the start of the test session were included, and the step-wise function was used to exclude non-significant predictors to ensure study power was not compromised by the large number of factors. All analyses were conducted using IBM SPSS 22 run on a Macintosh computer.

## Results

### Group characteristics

The overall age, BMI and scores on the TFEQ-D, TFEQ-R and TFEQ-H scales and rated hunger at the start of testing are shown in Table 2. There were no significant differences between cue conditions for any of these variables. In this study population, we also noted that TFEQ-D and TFEQ-R scores were not significantly correlated (r = 0.13, p = 0.20), and nor did TFEQ-D and TFEQ-H (r = 0.19, p = 0.06). None of the TFEQ subscales correlated significantly with BMI.

**Table 2:** Mean (±SEM) scores on the two scales of the TFEQ, the hunger rating at the start of testing and age and BMI depending on cue condition (Food or Control).

	Exposure condition		
	Food	Control	t-test
BMI (kg/m^2^)	22.7 ± 0.5	22.6 ± 0.5	t(95) = -0.14, p = 0.89
Age (years)	21.8 ± 0.4	21.0 ± 0.2	t(95) = -1.43, p = 0.16
TFEQ-D	7.1 ± 0.5	6.6 ± 0.5	t(95) = -0.74, p = 0.46
TFEQ-R	7.1 ± 0.7	8.1 ± 0.8	t(95) = 0.91, p = 0.37
TFEQ-H	6.2 ± 0.5	6.3 ± 0.5	t(95) = -0.06, p = 0.58
Rated hunger (100pt VAS)	35 ± 2	37 ± 3	t(95) = 0.55, p = 0.89

### DDT task

**Fig 2:**
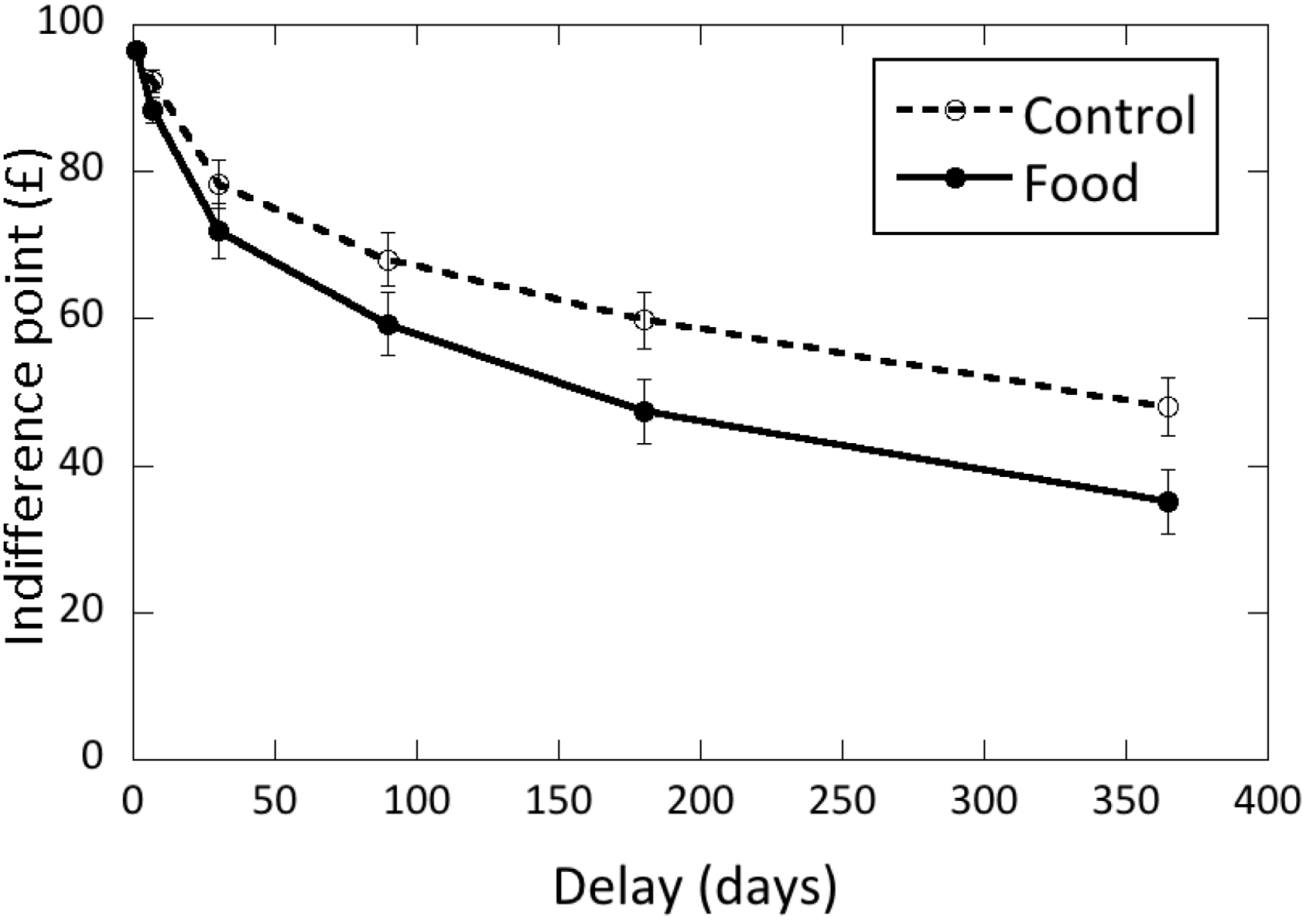
Mean (±SEM) indifference points on the delay-discounting task (DDT) as a function of delay to the larger reward in the Control (dashed line) and Food (solid line) conditions.

**Fig 3:**
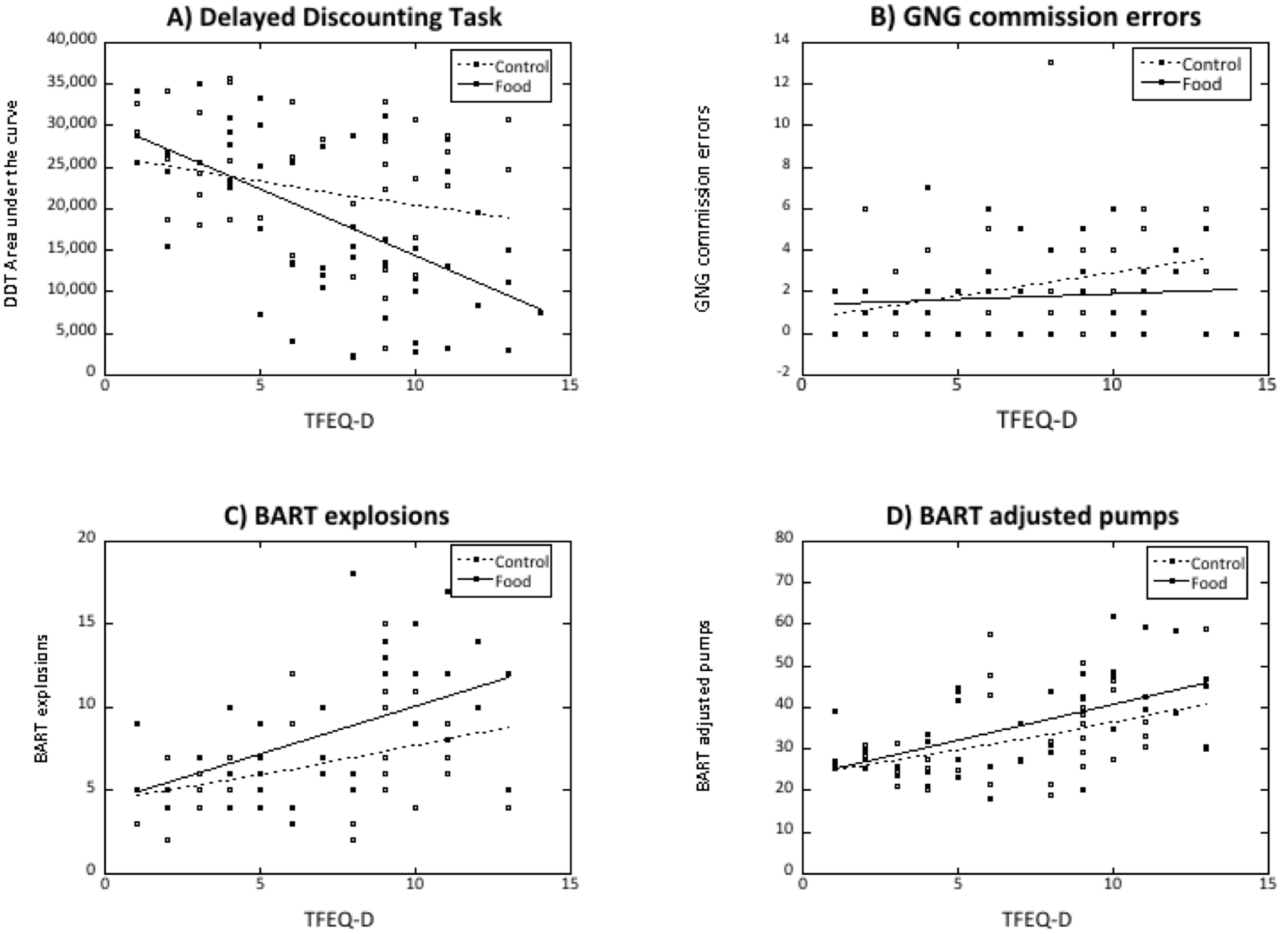
Key measures of impulsivity and risky decision in relation to TFEQ-D scores in the food (solid circles) and control (open circles) exposure conditions. Best-fit regression lines relating each measure to TFEQ-D scores in the two exposure conditions (food, solid line: control, dashed line). Measures shown are (A) DDT area under the curve, (B) commission errors from the GNG task, (C) explosions on the BART task and (D) adjusted average pumps on the BART task.

Overall indifference points on the DDT task at all six delays in the two cue exposure conditions are shown in Fig 2. Analysis of these data confirmed that indifference point decreased with delay as expected [F(5,460) = 15.23, p<0.001, ETA = 0.14], but this effect interacted with TFEQ-D score [F(5,460) = 9.25, p<0.001, ETA = 0.09], with TFEQ-D score having an overall significant effect on indifference scores [F(1,92) = 21.67, p < 0.001, ETA = 0.19]. There were also trends for a significant effect of exposure condition [F(1,92) = 3.42, p = 0.068, ETA = 0.04] and an interaction between exposure condition and delay [F(5,460) = 2.15, p = 0.059, ETA = 0.02]. No effects involving TFEQ-R approached significance in this analysis. To clarify these effects further, DDT scores were summarized as area under the curve (AUC) measures. Regression analysis of these DDT AUC data found a significant overall model [F(3,95) = 10.55, p<0.001], but only the interaction between TFEQ-D scores and condition was a significant predictor of DDT AUC (Beta = -0.75, t = 2.10, p = 0.038). To interpret this interaction, the regression was repeated separately for the two exposure conditions (Fig 3a). Following food cue exposure, DDT AUC decreased with increasing TFEQ-D scores [F(1,46) = 26.62, p<0.001: Beta = -0.60), whereas after exposure to the control stimuli there was no significant variation in DDT AUC as a function of TFEQ-D scores [F(1,46) = 2.27, p = 0.14, Beta = -0.22].

### Go-NoGo task

The key measure of impulsivity on the GNG is the number of commission errors. Regression modelling of these data found an overall significant model [F(2,94) = 6.71, p = 0.002], but there was no significant effect of cue exposure. Numbers of commission errors increased with TFEQ-D score Beta = 0.22, t = 2.26, p = 0.026: Fig 3b), and also increased with age (Beta = 0.24, t = 2.48, p = 0.015). Like commission errors, regression modelling of omission errors [F(1,94) = 13.16, p < 0.001) also found no effect of cue exposure but again found an effect of TFEQ-D (Beta = 0.35, t = 3.62, p < 0.001), with omissions increasing with TFEQ-D score. There were no significant effects of hunger, BMI or TFEQ-R, or interaction between cue condition and either TFEQ measure, in these analyses. In contrast, modelling of the reaction time measure [F(1,94) = 18.08, p < 0.001] found an effect of TFEQ-R (Beta = 0.40, p < 0.001), with slower response times as restraint increased, but no significant effects of cue exposure, TFEQ-D, hunger, BMI or age.

### BART task

In the regression analysis of the numbers of balloon explosions on the BART task [F(1,76) = 27.28, p < 0.001], the only significant predictor was the interaction between cue and TFEQ-D (Fig 3c). As with the DDT AUC measure, separate regressions were subsequently run for each cue condition to clarify this interaction. Both models were significant: food cue [F(1,38) = 14.74, p < 0.001], control cue [F(1,35) = 5.93, p = 0.02]. However the coefficient relating BART explosions to TFEQ-D tended to be larger after exposure to food (0.57 ± 0.15) than control cues (0.35 ± 0.14), although this difference was not significant (t(73) = 1.09, p = 0.28). The second measure, average adjusted pumps on each trial [36], also increased with TFEQ-D score [F(1,75) = 25.67, p < 0.001: Beta = 0.50, t = 5.09. p < 0.001), but this measure was unaffected by exposure condition (Beta = 0.14, t = 1.38. p = 0.17) nor was there any significant interaction between TFEQ-D and condition (Fig 3d). There were no significant effects of TFEQ-R or any other factor in these analyses.

### BIS/BAS questionnaire

**Fig 4:**
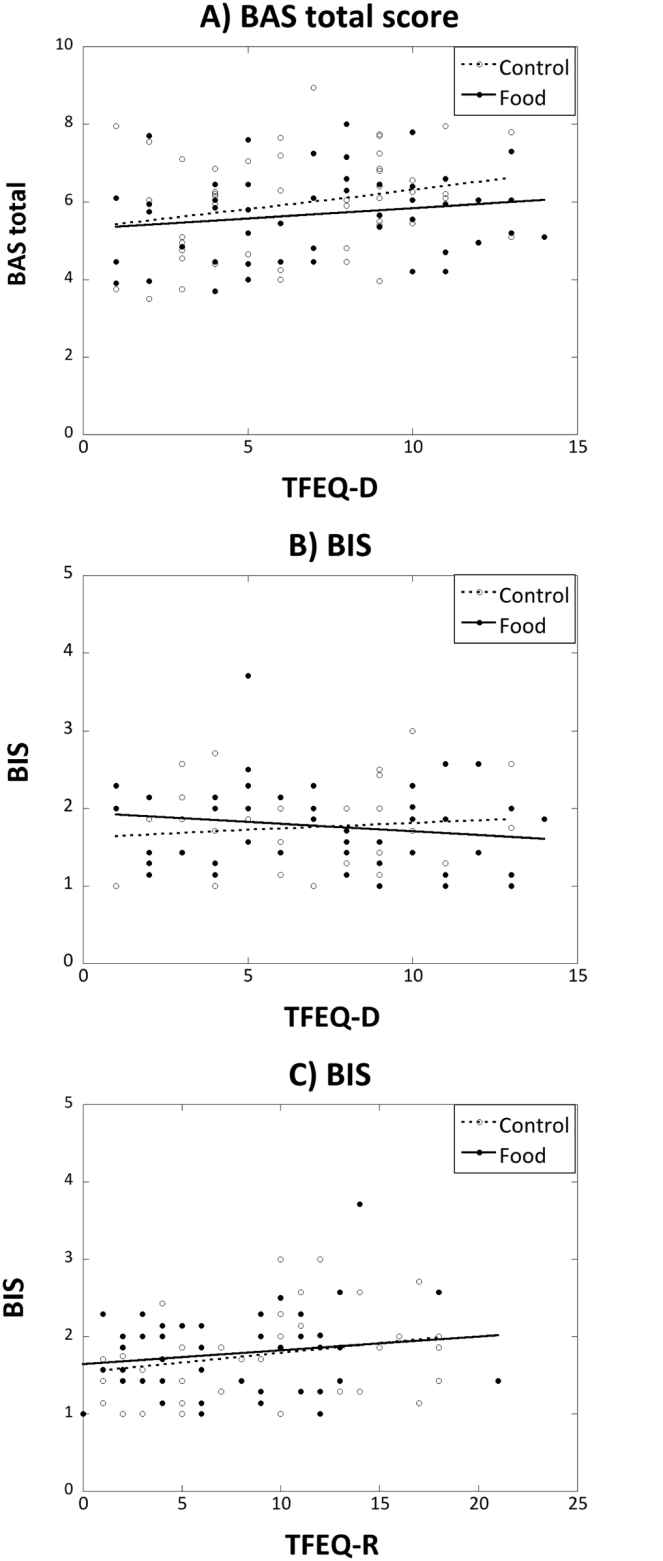
The relationship between (A) the BAS total and (B) BIS scores and responses on the TFEQ-D subscale, and (C) BIS scores related to scores on the TFEQ-R subscale, in the two exposure conditions (food, filled circles and solid line: control, open circles and dashed line)

Regression analyses of overall scores on the BAS (Fig 4a), as well as the three BAS sub-scales (data not shown), did not find any significant predictor of BAS scores, although TFEQ-D tended to correlate with the BAS total score (r(95) = 0.20, p = 0.052). In contrast, scores on the BIS increased significantly with TFEQ-R [F(1,94) = 4.34, p = 0.04, Beta = 0.21, t = 2.08, p = 0.04, Fig 4c], but were unaffected by TFEQ-D or exposure condition (Fig 4b).

## Discussion

The key finding from this study was the first evidence we are aware that pre-exposure to food cues resulted in an increase in impulsive choice measured using the DDT and risky behaviour measured by the BART task for women who scored higher on the TFEQ-D scale, and also confirmed that higher scores on TFEQ-D were associated with greater impulsivity measured by the DDT, risk taking measured using BART and impulsive responding on the GNG. In contrast, restrained eating was not associated with impulsive responding on the DDT, BART or GNG, and was unrelated to the effect of cue exposure on performance on any of the tasks. Restrained women did score higher on the BIS scale. The key finding was thus that young women whose TFEQ-D scores suggested that they were prone to uncontrolled eating showed a heightened tendency to act impulsively in general but this was tendency was enhanced by prior exposure to food cues.

The present data further confirm that self-reported tendencies to show uncontrolled eating in normal-weight women are related to some aspects of impulsivity and risky decision making. Women scoring higher on the TFEQ-D showed greater impulsive choice overall, replicating our earlier finding [20], but this effect was greater when the DDT was administered after seeing a series of food cues than seeing visually-matched control stimuli. More recently we only found a weak (non-significant) correlation between DDT performance and TFEQ-D scores [19], but in that study participants were not tested in the presence of food cues. In the present data, the relationship between TFEQ-D score and DDT performance in the absence of food stimuli was also not significant. Thus behavioural impulsivity was triggered by prior exposure to a relevant reward: the sight of food by women prone to uncontrolled eating. This finding suggests that reward-related behavioural acts of impulsivity are more likely if primed with a relevant reward. This effect was not limited to the DDT, however, as we also found that food-cue exposure lead to more risky behaviour on the BART task, the first time that we are aware of differences in eating being related to BART performance. There are parallels between the present findings and the results of a study examining how DDT performance interacts with cue exposure to enhance intake of palatable foods [34]. Although in that study everyone was exposed to food cues in the absence of hunger, and the possibility that food cues might have enhanced responding on the DDT by reward-sensitive participants could not be tested, the finding that those participants who were food-reward sensitive (measured by the Power of Food Scale) and who were impulsive on the DDT consumed more palatable food bears similarity to the individual differences in enhanced DDT performance after exposure to food reported here, especially as the TFEQ-D and Power of Food measures have been found to correlate [43].

Whereas performance on both the DDT and BART were affected by cue exposure, performance on the GNG task was not. Numbers of commission errors on the GNG were associated with TFEQ-D scores, however, numbers of such errors were low and the task may have lacked the sensitivity to pick up effects of the food-cue manipulation. Thus it should not be concluded that effects of cue exposure do not impact impulsive action, but rather that the present study found no evidence that this was so. Indeed a recent related study reported that individuals defined as impulsive based on their responses to the Barratt impulsiveness scale who had been acutely exposed to food cues showed weaker inhibitory control on a Stop signal task than after exposure to non-food cues [44]. As scores on the TFEQ-D and Barratt impulsiveness scale have been found to correlate [20,45], these data further imply a lack of sensitivity in the GNG used here.

While some behavioural measures of impulsivity were moderated by cue-exposure, responses on the BIS/BAS were unaffected, confirming the trait nature of this questionnaire. Our finding of higher scores on the BIS as a function of restraint replicates an earlier finding [35], although a more recent study did not find this relationship [46]. We also found a weak (marginally significant) correlation between the overall BAS score and TFEQ-D (r = 0.20) which concords with a small non-significant correlation between these measures in a larger sample (r = 0.13: [46]).

It might have been expected that food cue exposure would increase impulsivity in women as a function of dietary restraint, particularly since studies have shown that restrained eating interacts with impulsivity (measured using a Stop-signal task) to solicit overeating of palatable foods [47]. However, the only measures affected by restraint were the BIS, where the positive association could be interpreted as consistent with a general restrained habit, and reaction times on the GNG, where restrained women were slower, replicating a previous GNG study [48] and our recent study using a Stop-signal task [19]. Why restrained women were slower on these tasks is not clear, but neither of these results suggest a breakdown of restraint leading to impulsive responding in the way the impulsivity moderates responses to food cues to cause overeating.

Previously, studies found differences in how impulsivity impacted eating or food choice dependent on hunger-state [32]. It might then have been expected that hunger state at the time of testing in the present study would have modified impulsive responding, but there was no evidence that this was so, with no effect of hunger state on any of the impulsivity measures and hunger did not interact with cue exposure to enhance impulsive responding. However, the previous study specifically manipulated hunger state whereas the present study relied on natural variation, and it may be that more explicit food deprivation would have found evidence of an effect of hunger on cue-potentiated impulsivity.

How then might exposure to food cues enhance impulsive responding on the DDT and risk-taking on the BART? The present data cannot offer a mechanistic explanation but other data suggest at least two possibilities based around evidence that two key brain areas, the striatum and orbitofrontal cortex (OFC), are implicated in the relative preference for immediate and delayed rewards [49,50]. The first idea is that food cues enhance reward value in the striatum, and this enhanced reward value then modifies response in the BART and DDT tasks. Dopamine activity in the ventral striatum is strongly associated with reward reactivity [51]. Neuroimaging studies in humans have shown increased activation of the striatum during anticipation of reward [52]. Cues that predict reward have been shown to enhance striatal dopamine release. For example, in animal studies cues that were associated with cocaine administration selectively increased dopamine release in the core of the nucleus accumbens [53], and dopamine release is necessary to elicit a reward-seeking behavioural response in rats [54]. Thus one suggestion is that exposure to food cues enhanced striatal dopamine and this underlies the enhanced impulsive responding. However, this would only work if the effects of exposure resulted in changes in dopamine signalling that were sustained over 20–30 minutes, which seems less plausible. The second suggestion relates to the known role of the OFC in impulsivity. Data from studies in animal models [55–57] and humans [58,59] both implicate the OFC as a key structure in determining preference for delayed rewards on the DDT, and damage to the OFC greatly increases the preference for immediate rewards [60]. Given the broader importance of the OFC in key aspects of appetite control and taste preference [61], and the acute activation of the medial OFC when viewing food pictures [11], OFC stimulation through exposure to food pictures may have residual effects in reward processing that increases the preference for immediate rewards on the DDT. Although both these ideas are speculative, they do open the way to future research exploring the neural basis of cue-enhanced impulsivity.

We recognize the preliminary nature of the reported findings, and there are a number of issues that need to be examined further. Firstly, although we tried to match the food and control cues on visual appearance, this was done by visual inspection (in line with most work using food pictures). The recent development of a more standardised library of food pictures [62] suggest that it would be valuable to repeat this study using pictures matched more closely in colour, intensity, etc., although it is very unlikely that the images we used would varied systematically in a way that could explain the specific impact of cue exposure on impulsivity reported here. Related to this, our food picture set included items that could be considered healthy and unhealthy. The rationale for including both healthy and unhealthy items was to reduce possible demand effects from repeated exposure to unhealthy foods, which could have lead participants in the food exposure condition to question the study cover-story. With hindsight this limits interpretation, and a follow-up study should examine these separately, using a more objective measure of healthy. Thirdly, the present study only used a limited battery of impulsivity tests, and given our recent finding that a measure of reflection impulsivity was the sub-type with the strongest association with TFEQ-D scores [19], follow-up studies need to examine a wider range of impulsivity measures. Fourthly, we used a fixed order of testing for the behavioural tasks, with the GNG completed immediately after the cue-exposure. However, we note that cue-exposure affected both DDT and BART performance even though these were completed several minutes after the cue exposure, and it is possible the memory-task instructions kept these cues in mind for longer. Finally, we would view the GNG outcome with caution as the task may have been too easy for the target population: a repeat with a more demanding GNG would be worthwhile. However, none of these limitations invalidate the key findings of the present study.

In summary, in this preliminary investigation, women scoring high on the TFEQ-D scale were more impulsive overall on the DDT and BART tasks, and made more commission errors on the GNG task but they acted more impulsively on the DDT and BART after exposure to food cues than did matched controls. In contrast, scores on the TFEQ-R were associated with higher scores on the BIS and slower performance on the GNG task, and were unaffected by cue exposure. These data tentatively suggest that the tendency for uncontrolled eating suggested by high TFEQ-D scores may relate to enhanced reward-reactivity which can be exacerbated by exposure to cues predictive of food reward.

## Supporting Information

S1 File The Powerpoint file used in the food cue condition. (PPTX)

S2 File The Powerpoint file used in the control cue condition. (PPTX)

S1 Dataset The full dataset used for analyses (Excel version). (XLSX)
