# Supplementary material for: Cued to Act on Impulse: More Impulsive Choice and Risky Decision Making by Women Susceptible to Overeating after Exposure to Food Stimuli
Source: PLoS One. 2015 Sep 17;10(9):e0137626. doi: 10.1371/journal.pone.0137626 (PMC4574976; doi:10.1371/journal.pone.0137626)
Supplement: S1 File — (PPTX) [file pone.0137626.s001.pptx]

## Slide 1
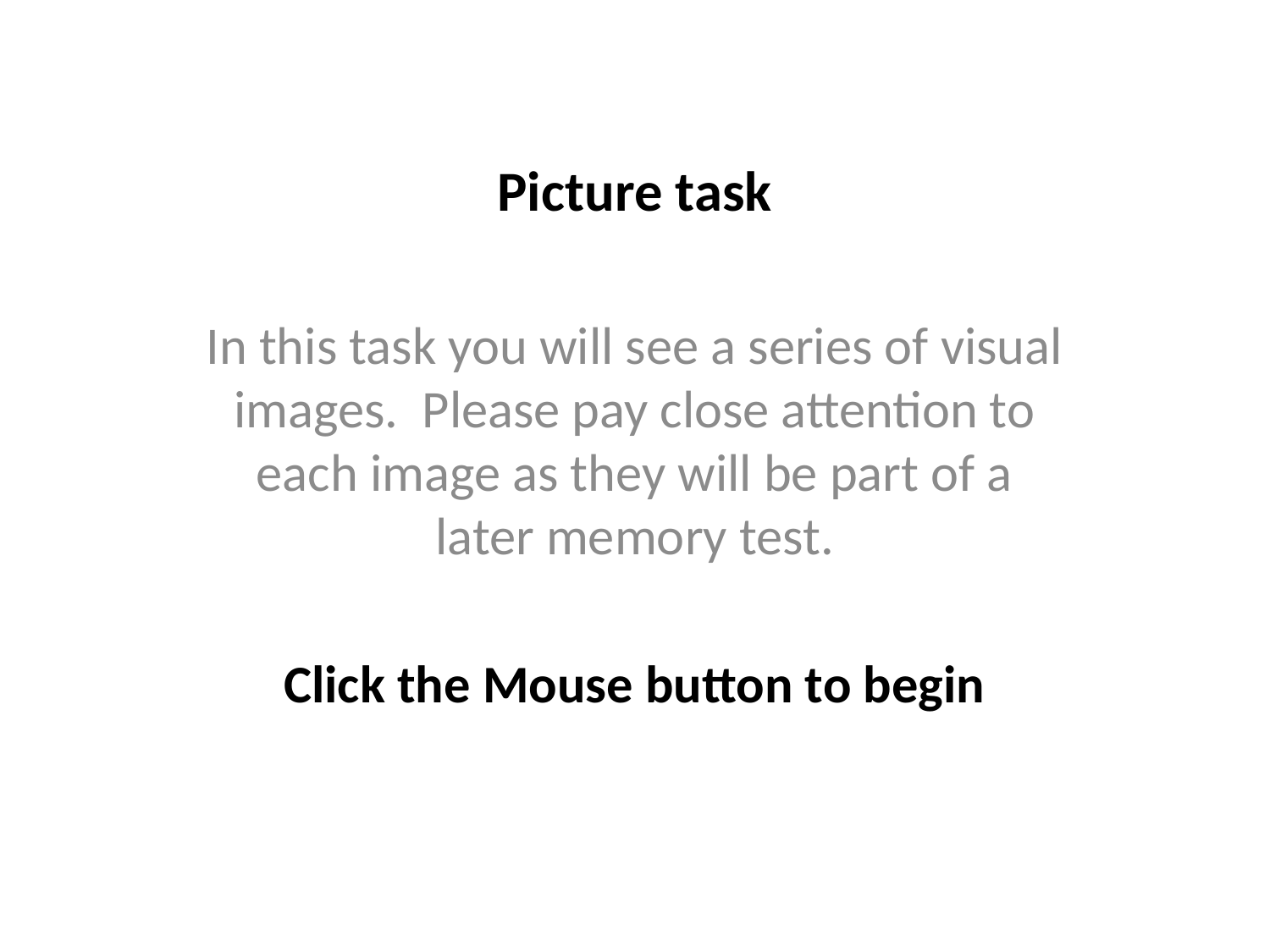

Picture task
In this task you will see a series of visual images. Please pay close attention to each image as they will be part of a later memory test.
Click the Mouse button to begin

## Slide 2
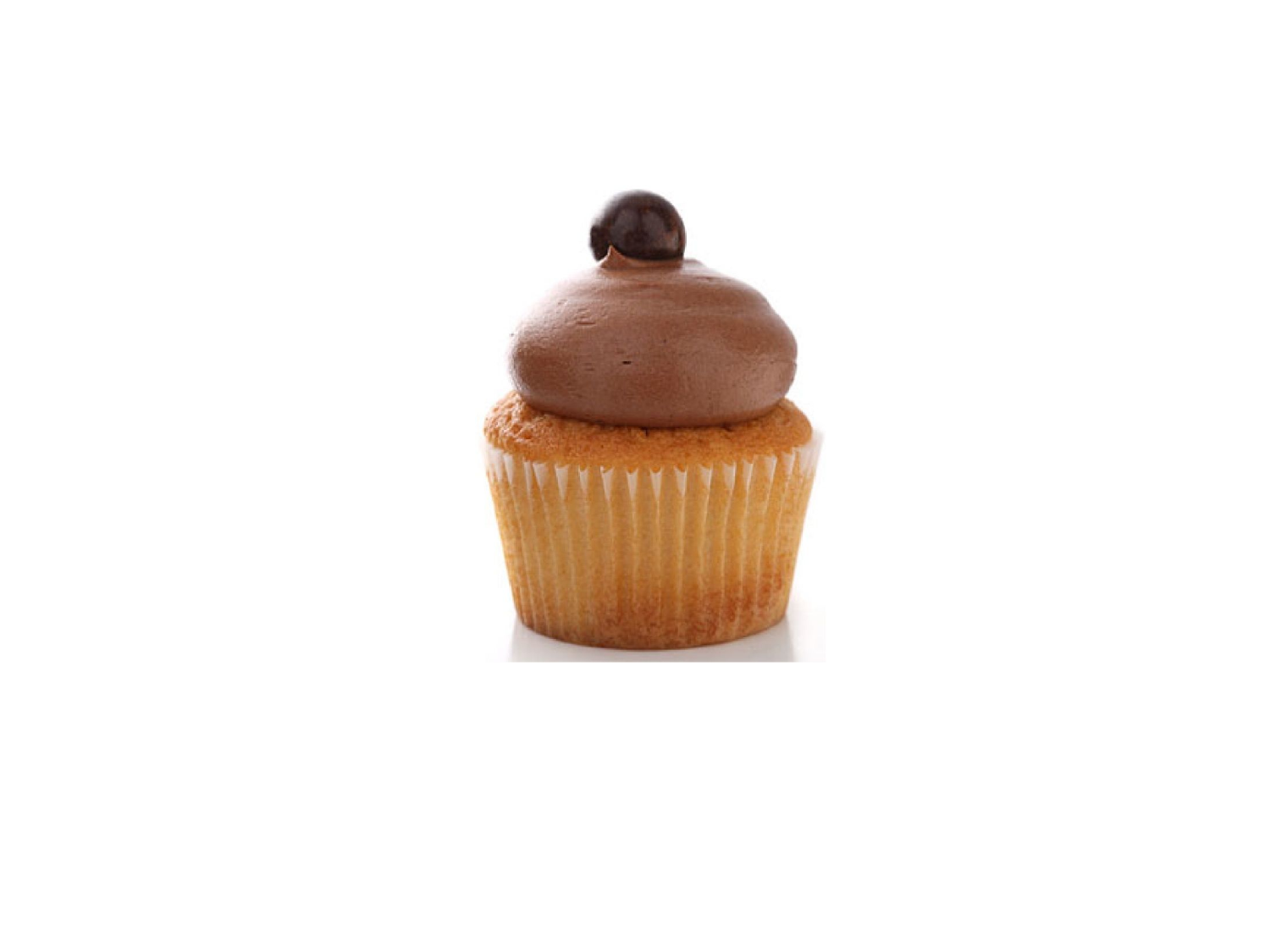

## Slide 3
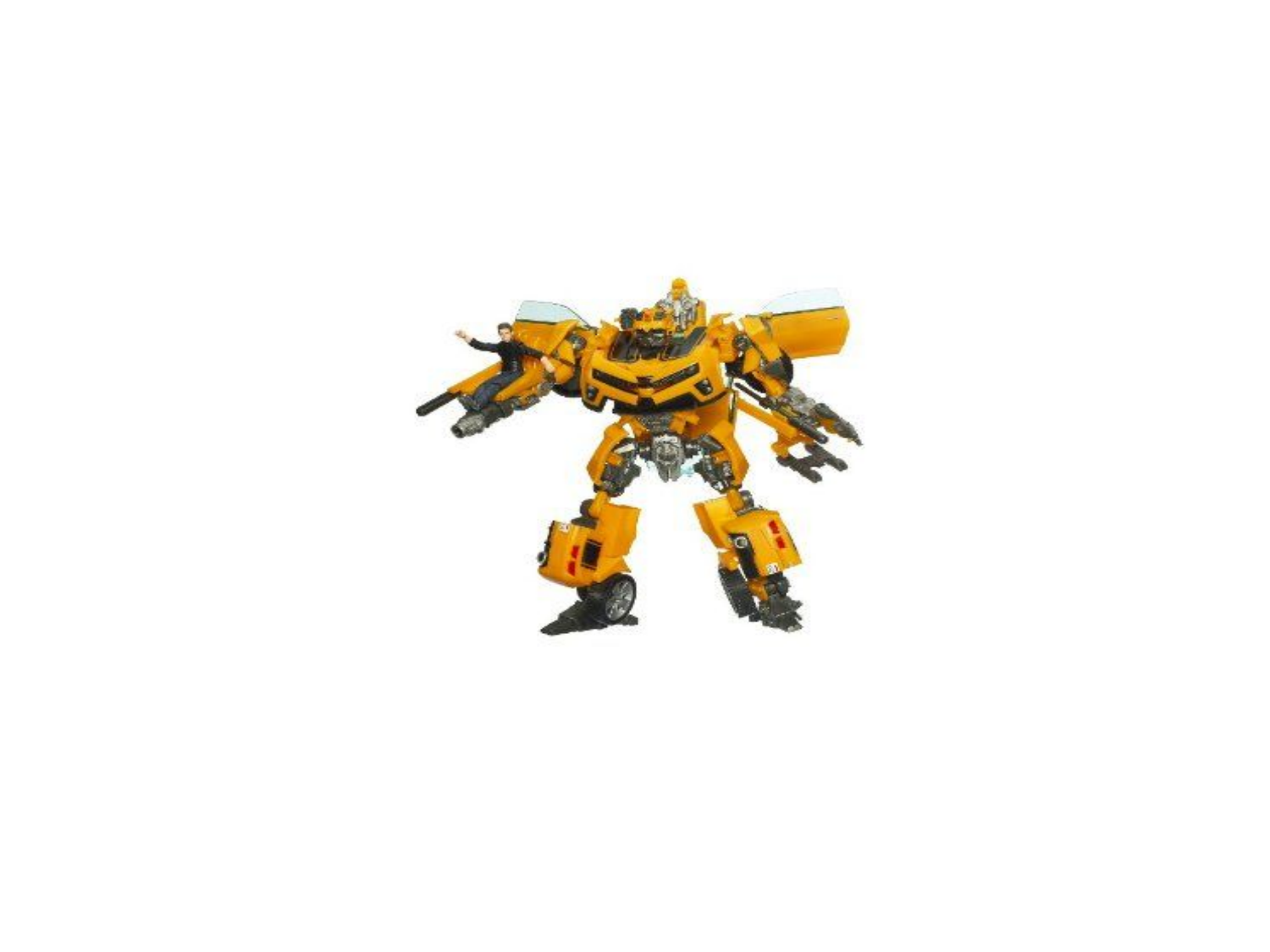

## Slide 4
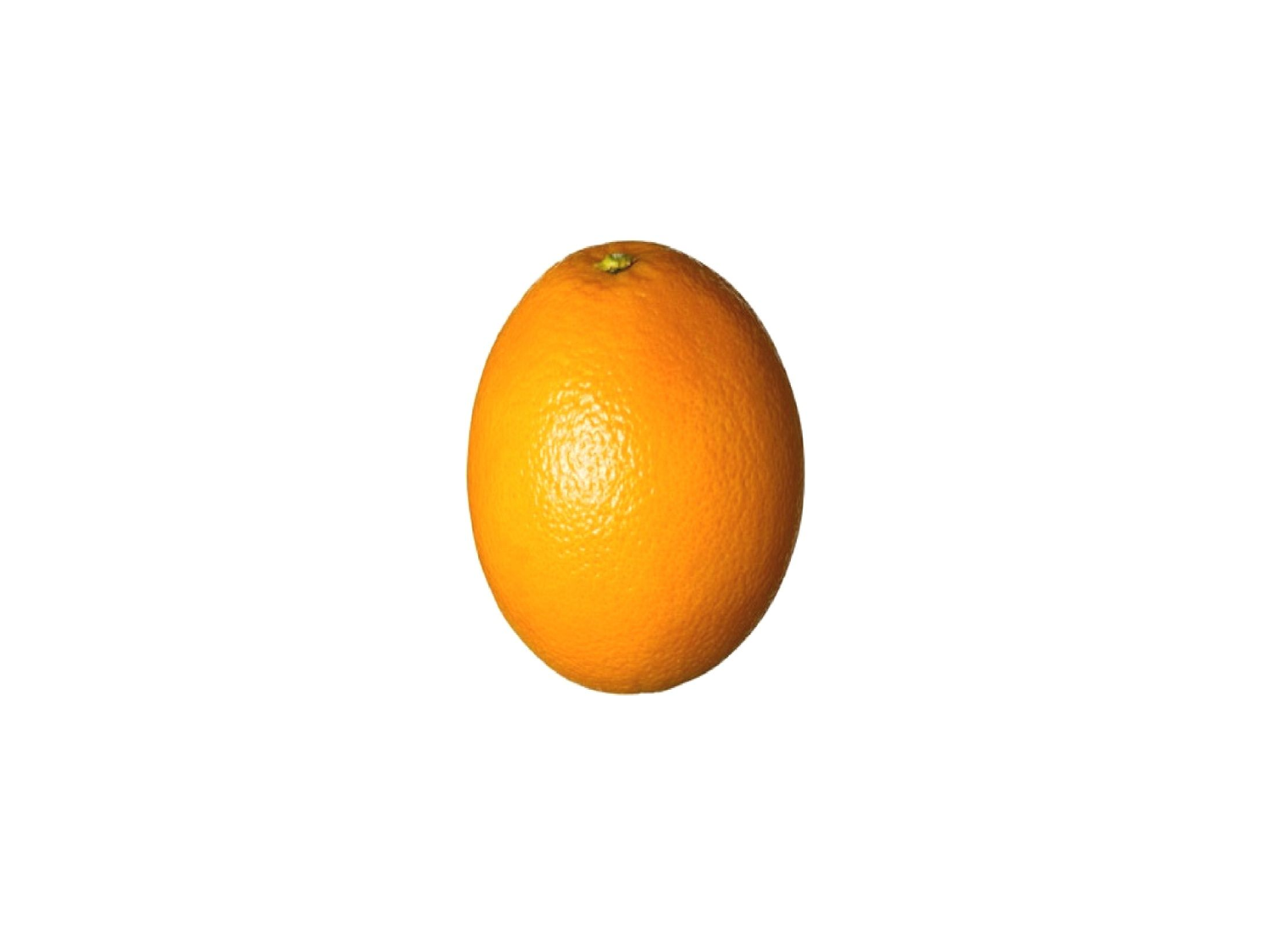

## Slide 5
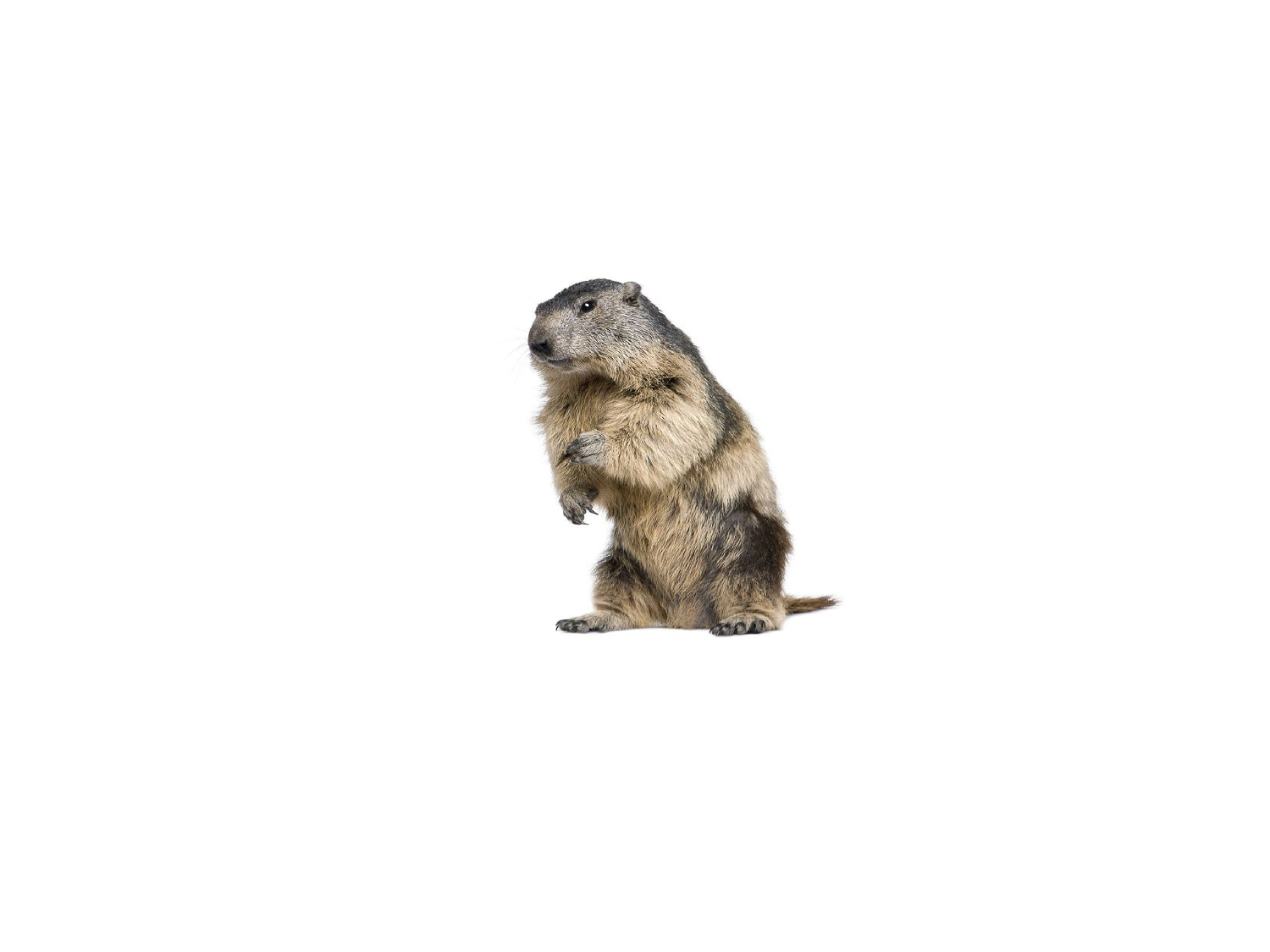

## Slide 6
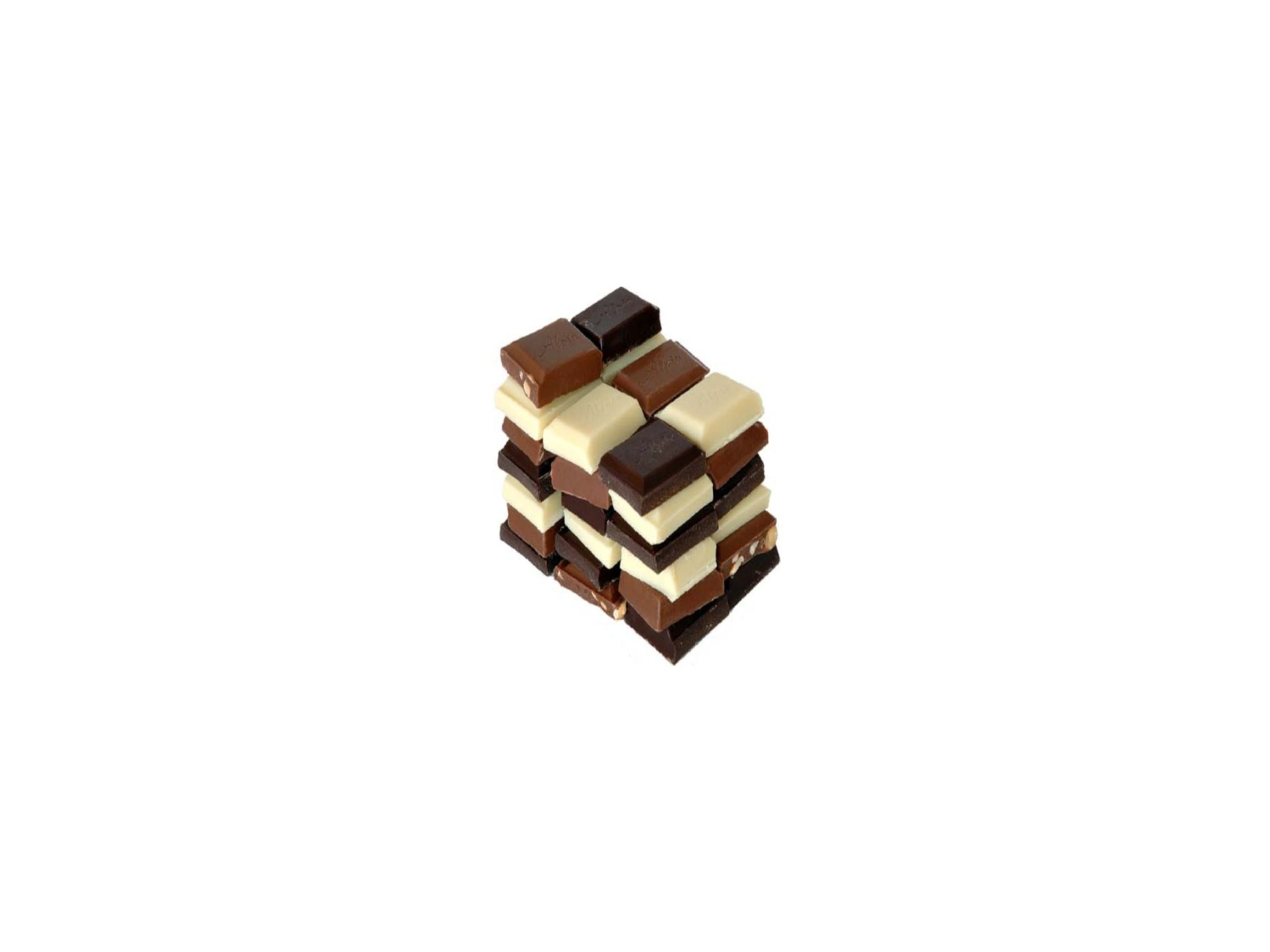

## Slide 7
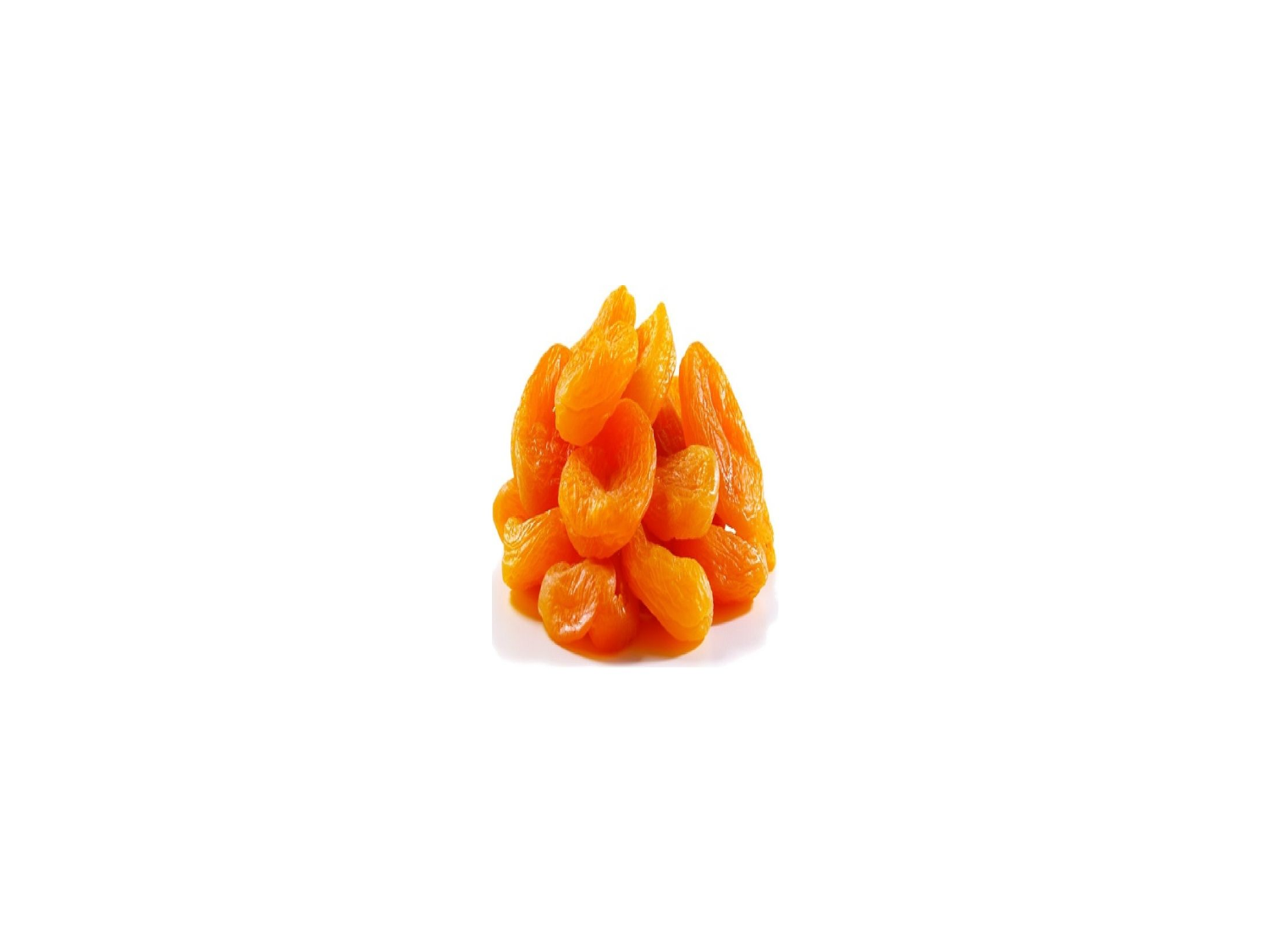

## Slide 8
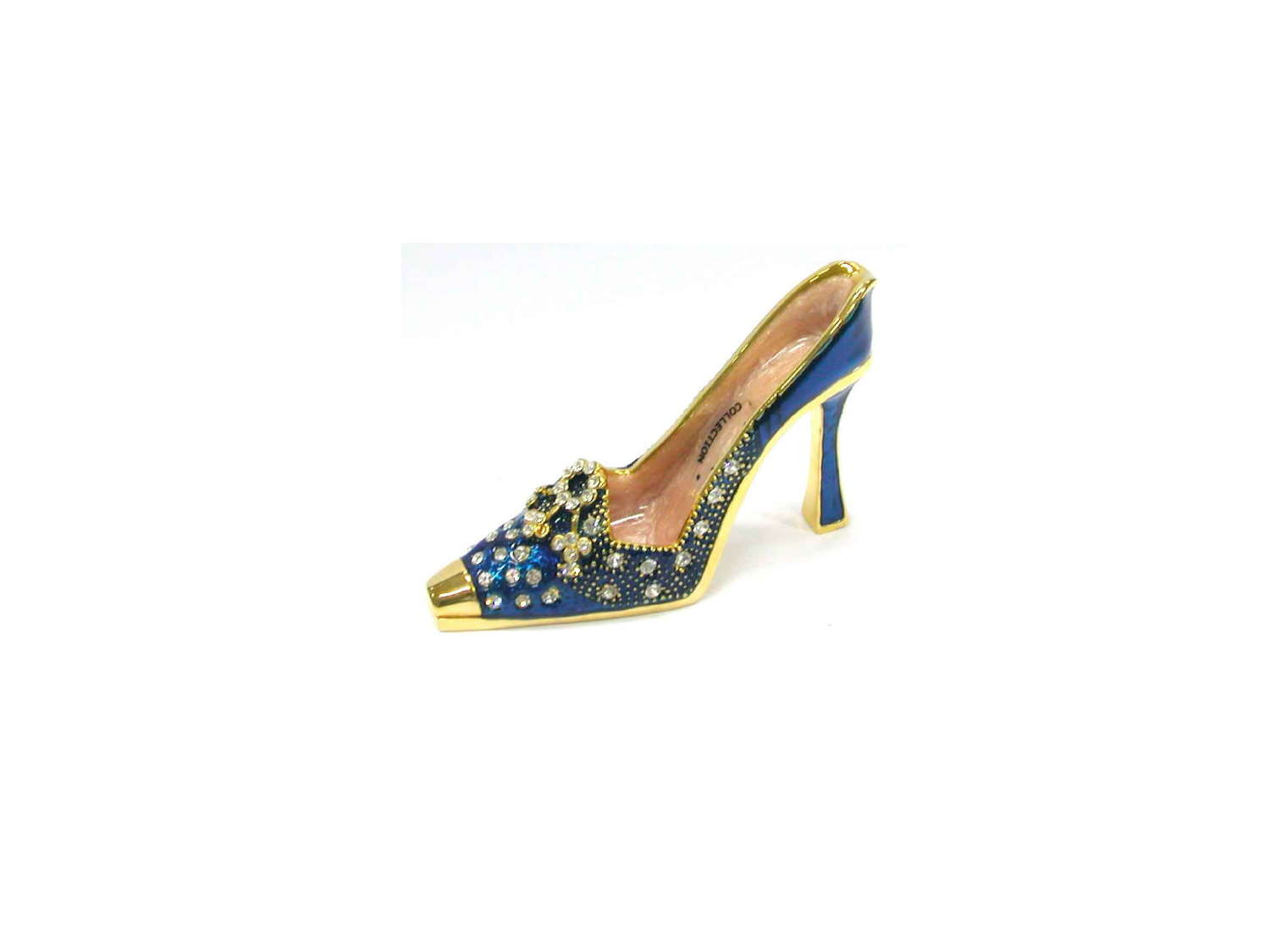

## Slide 9
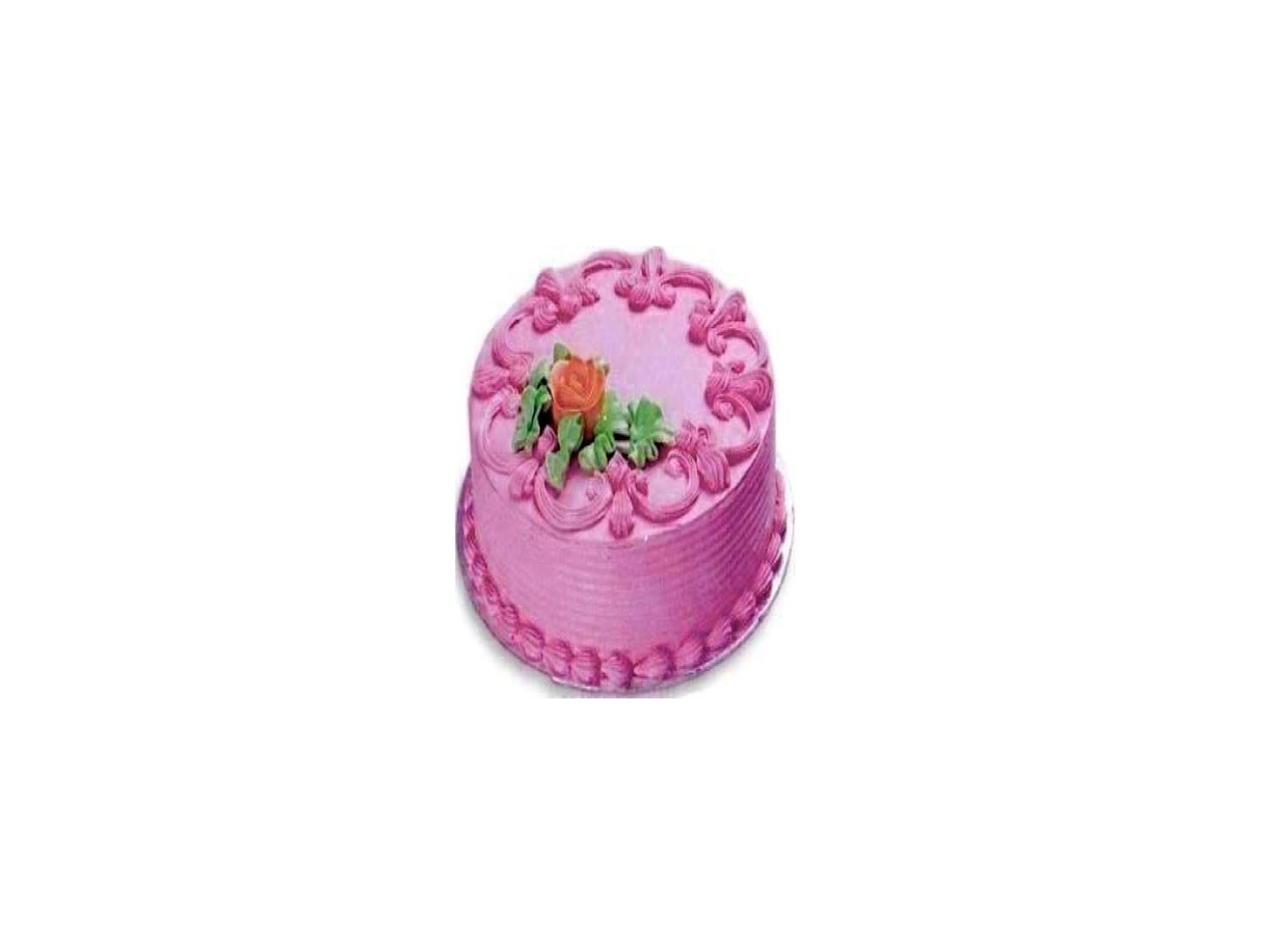

## Slide 10
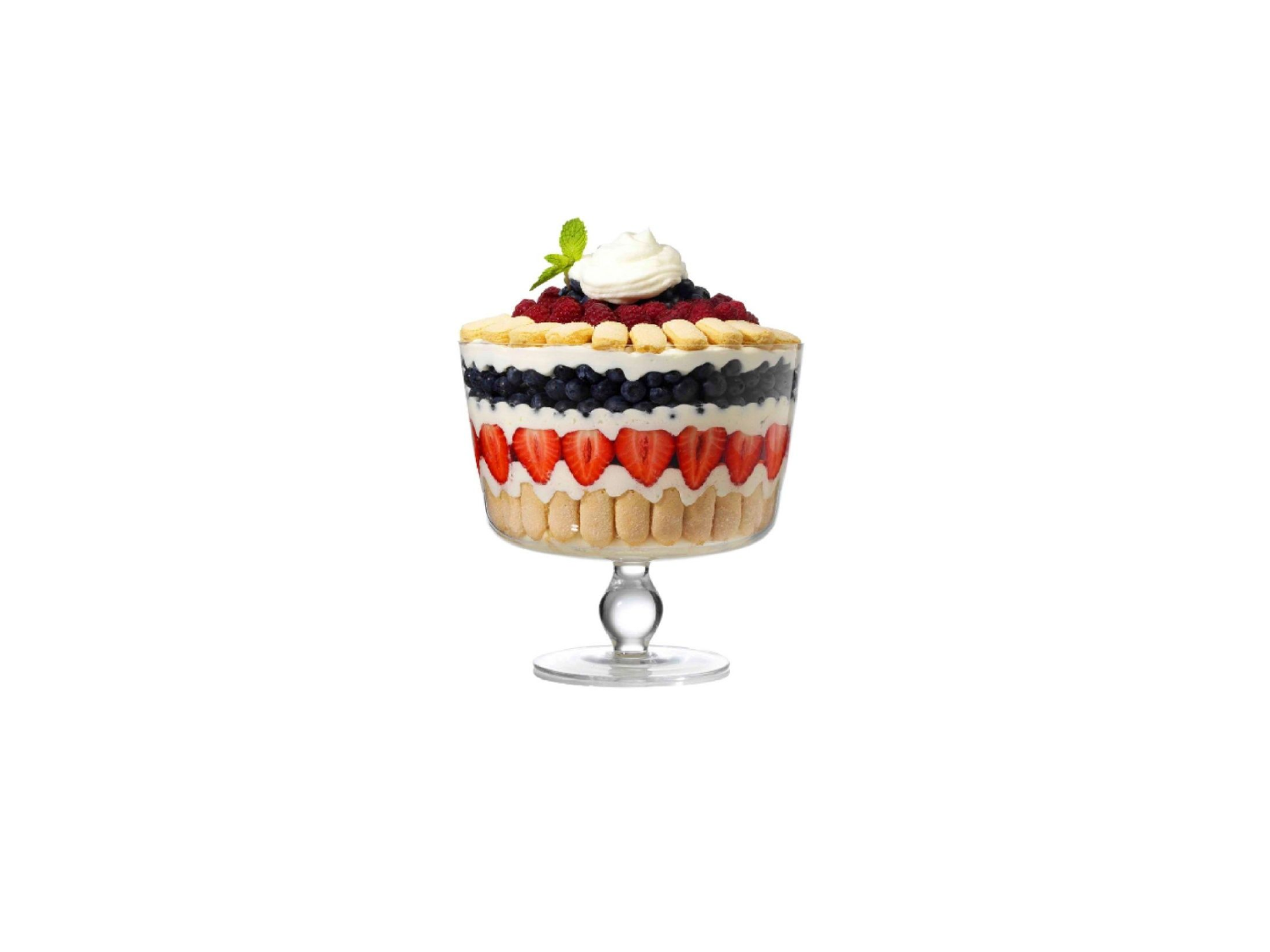

## Slide 11
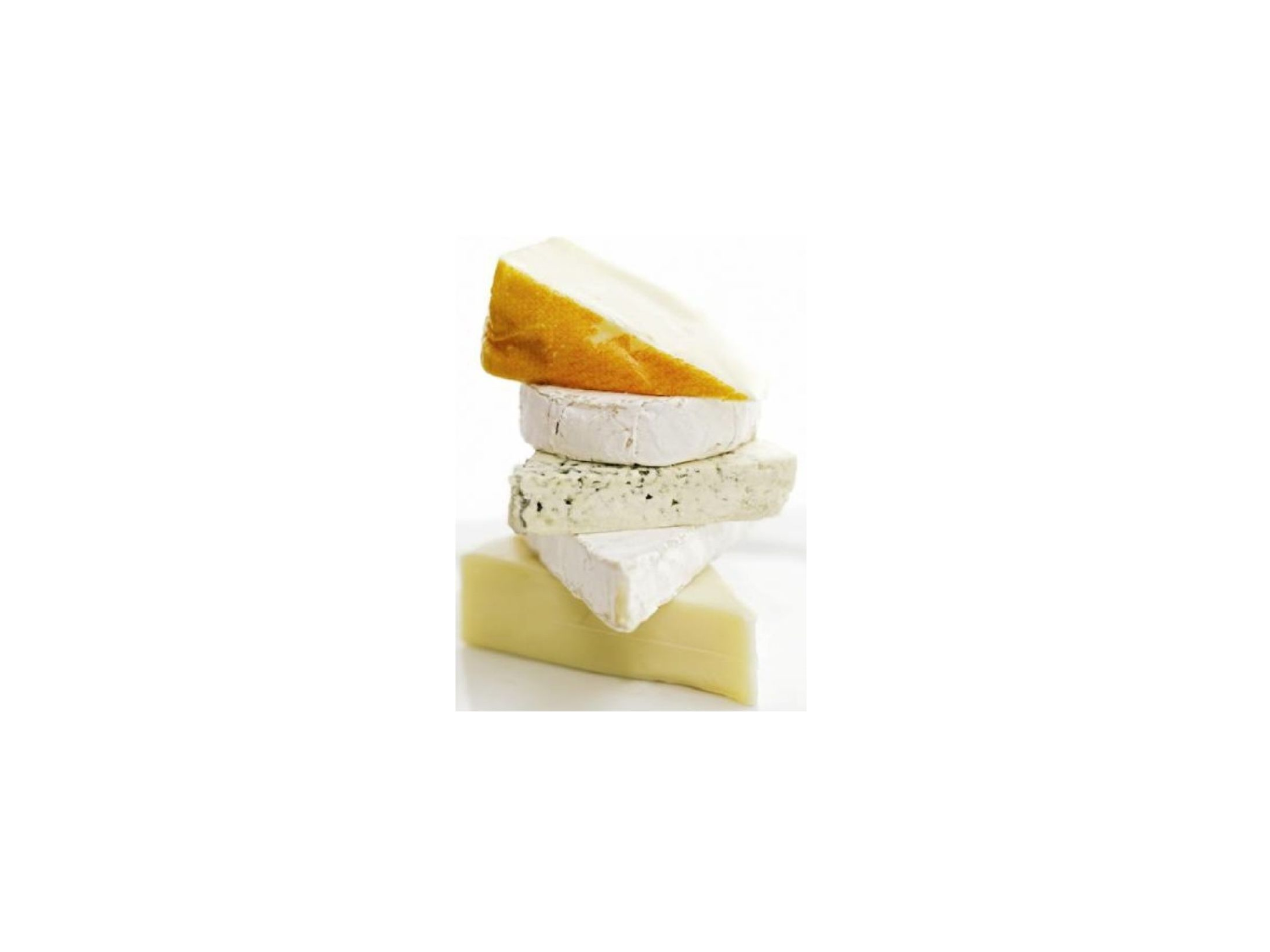

## Slide 12
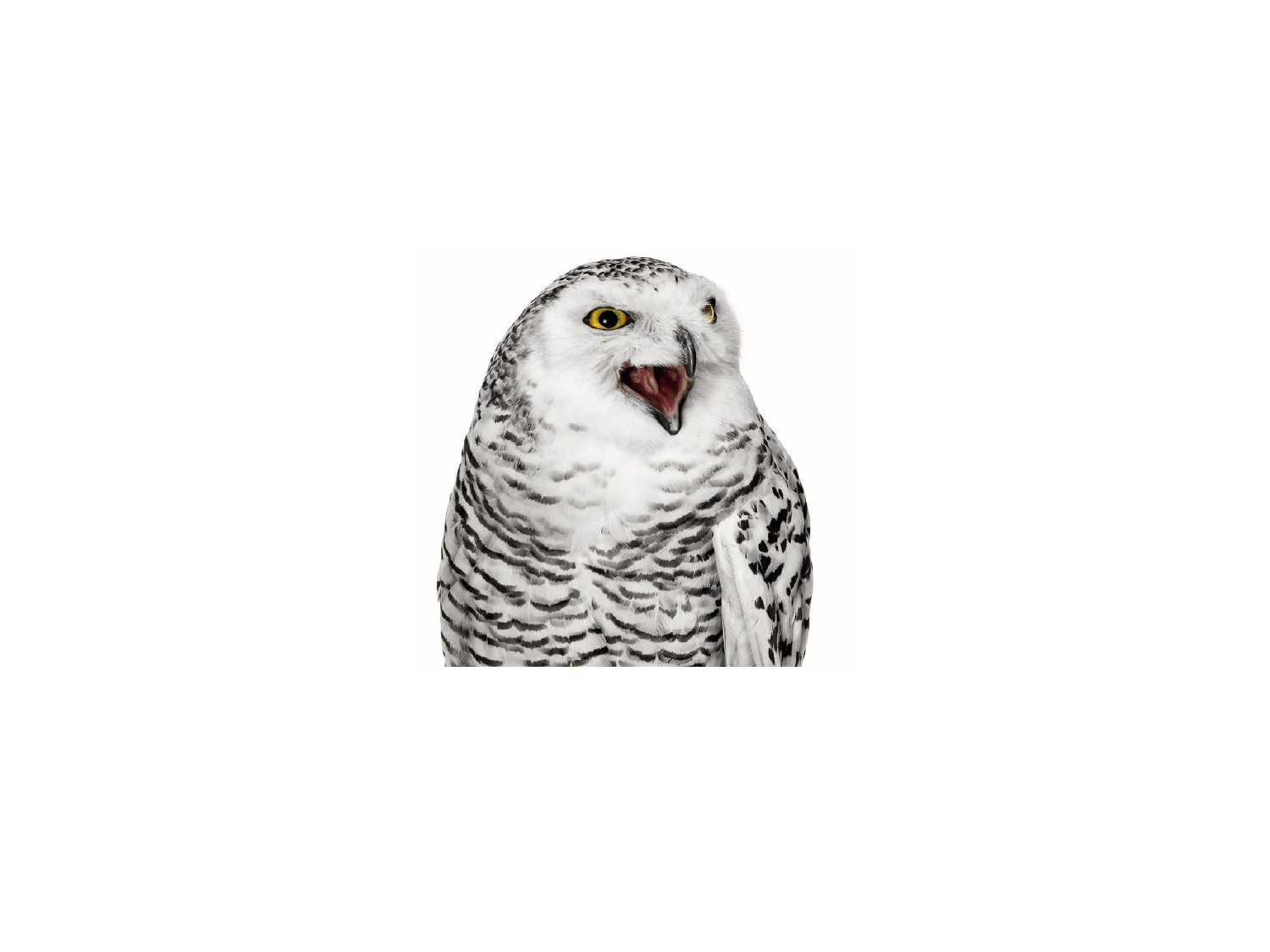

## Slide 13
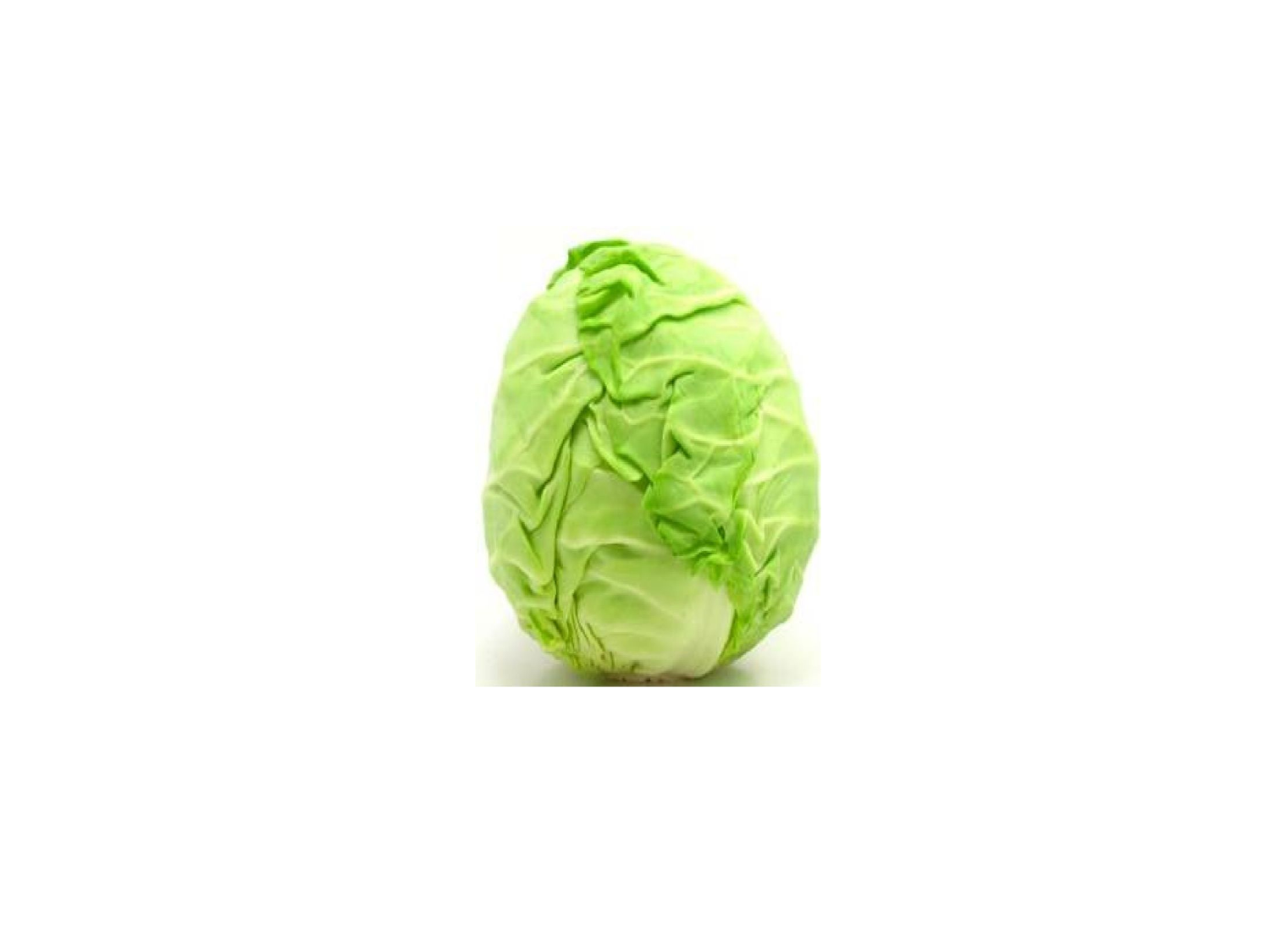

## Slide 14
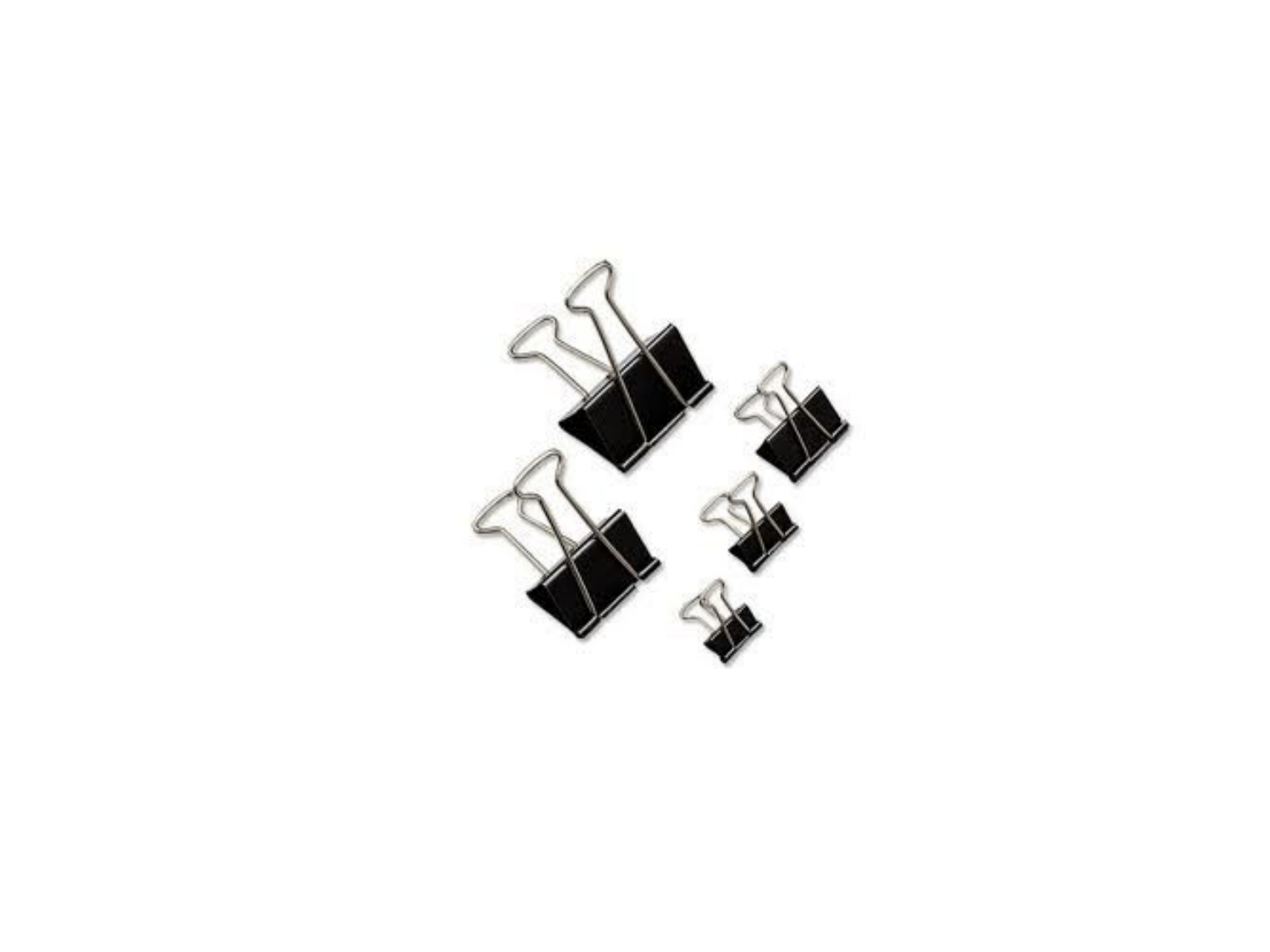

## Slide 15
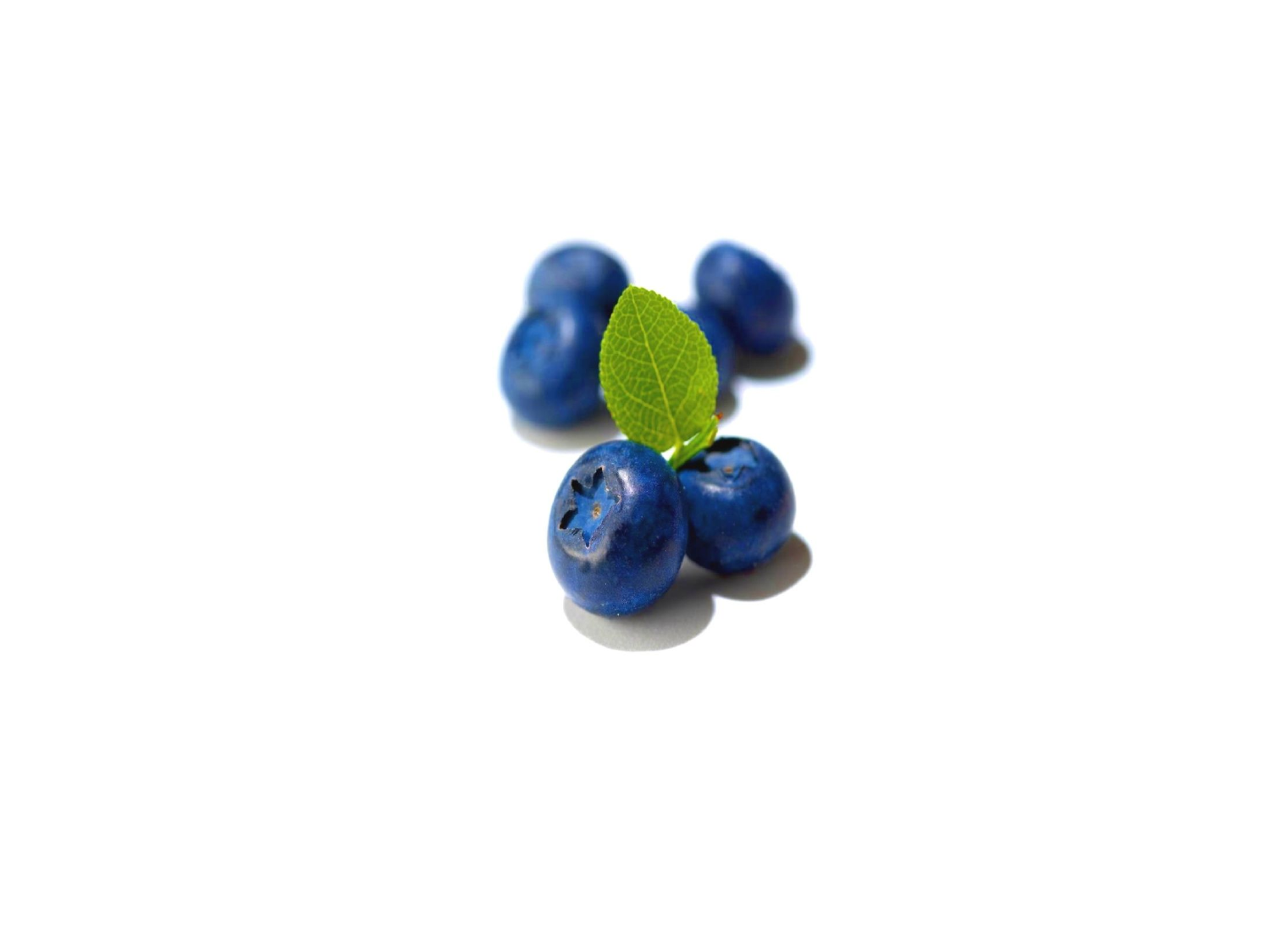

## Slide 16
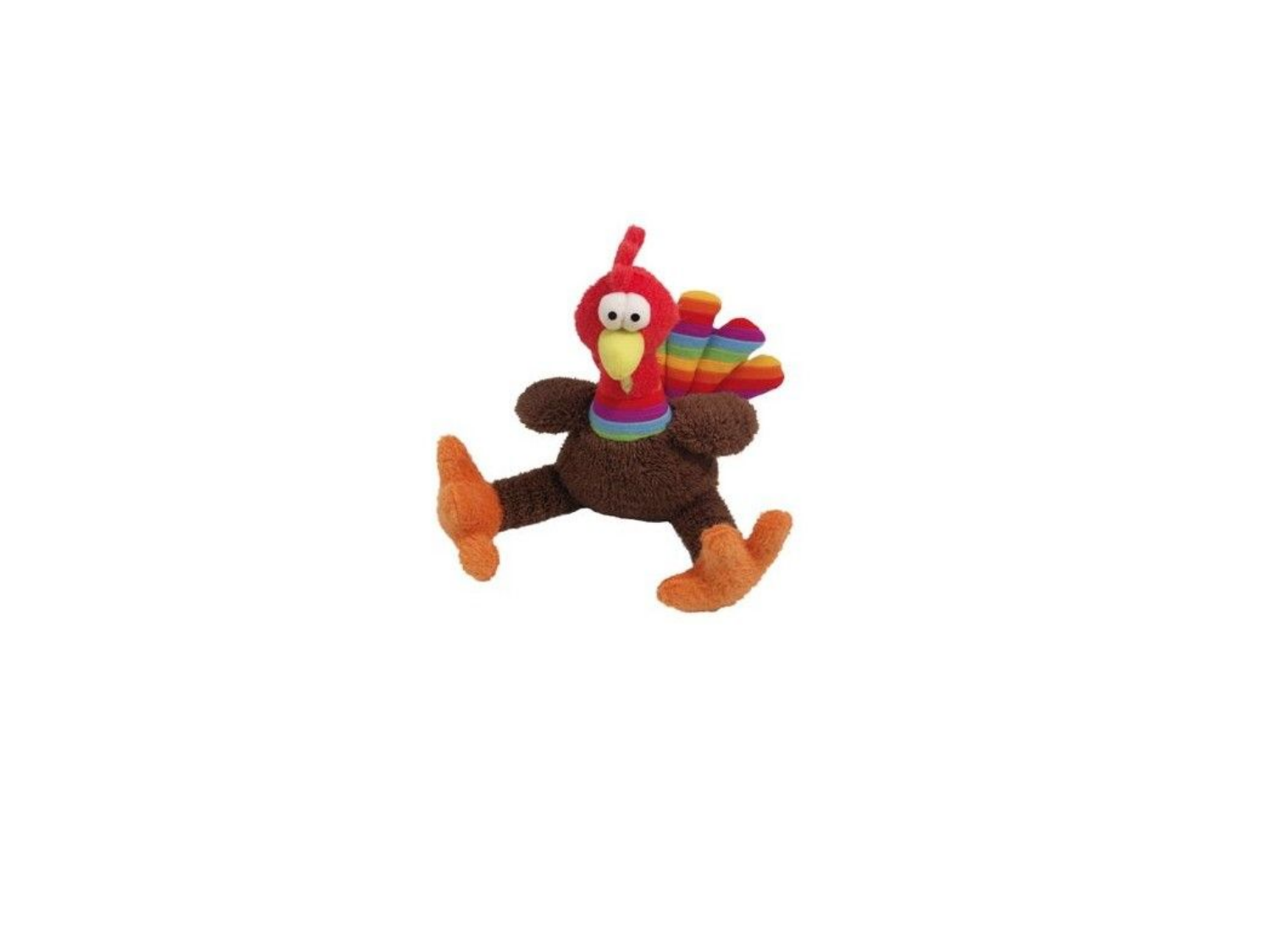

## Slide 17
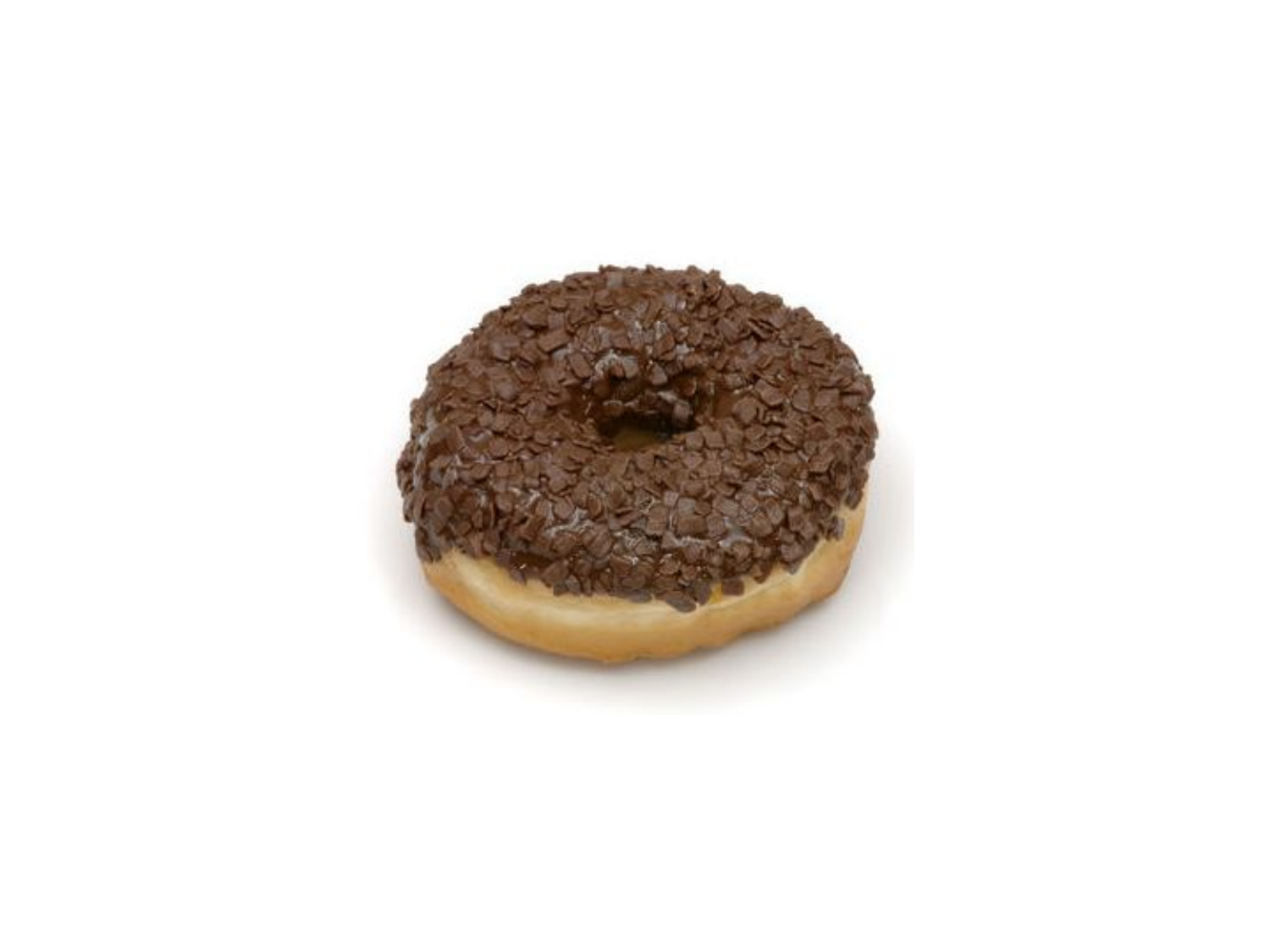

## Slide 18
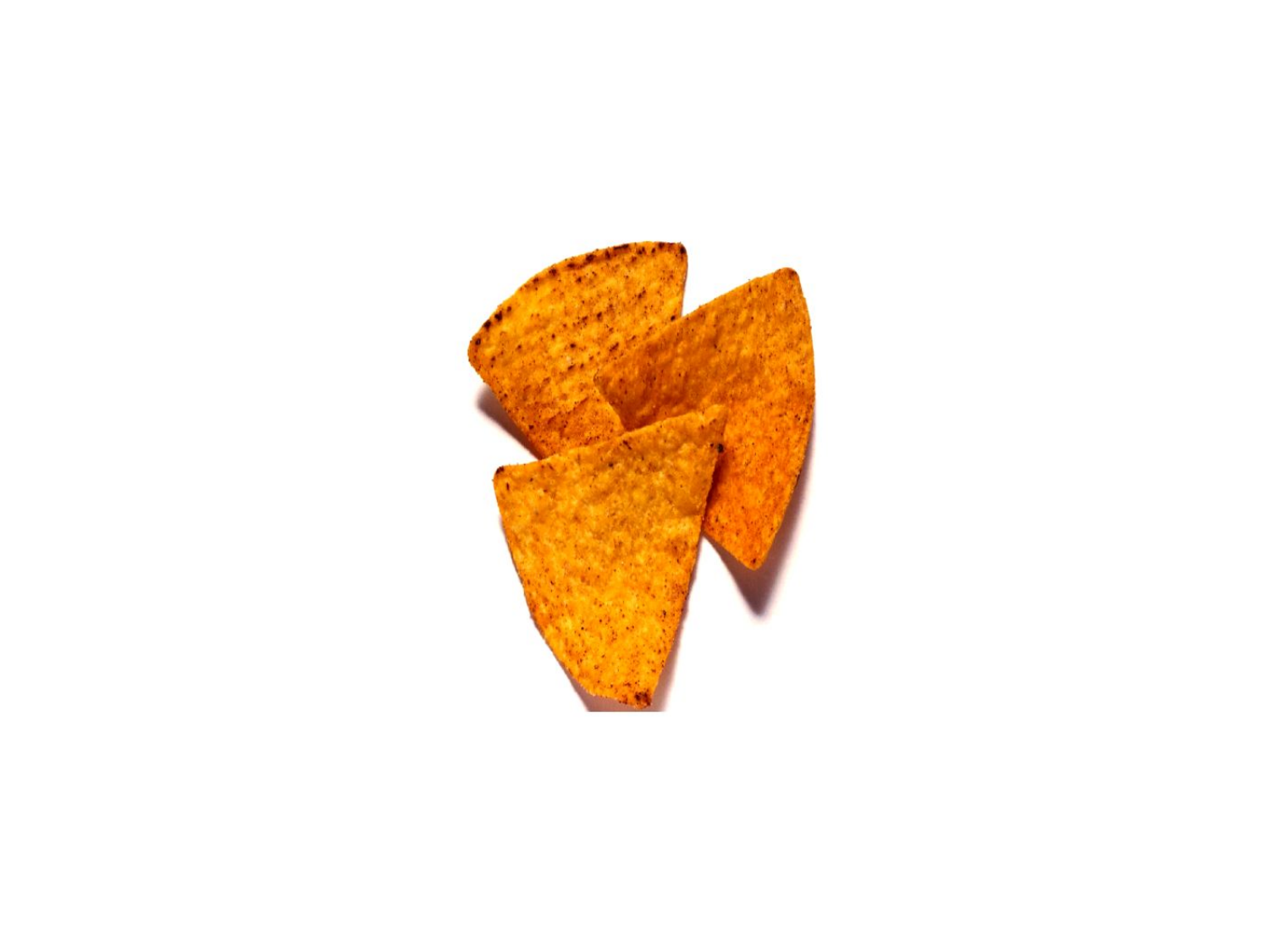

## Slide 19
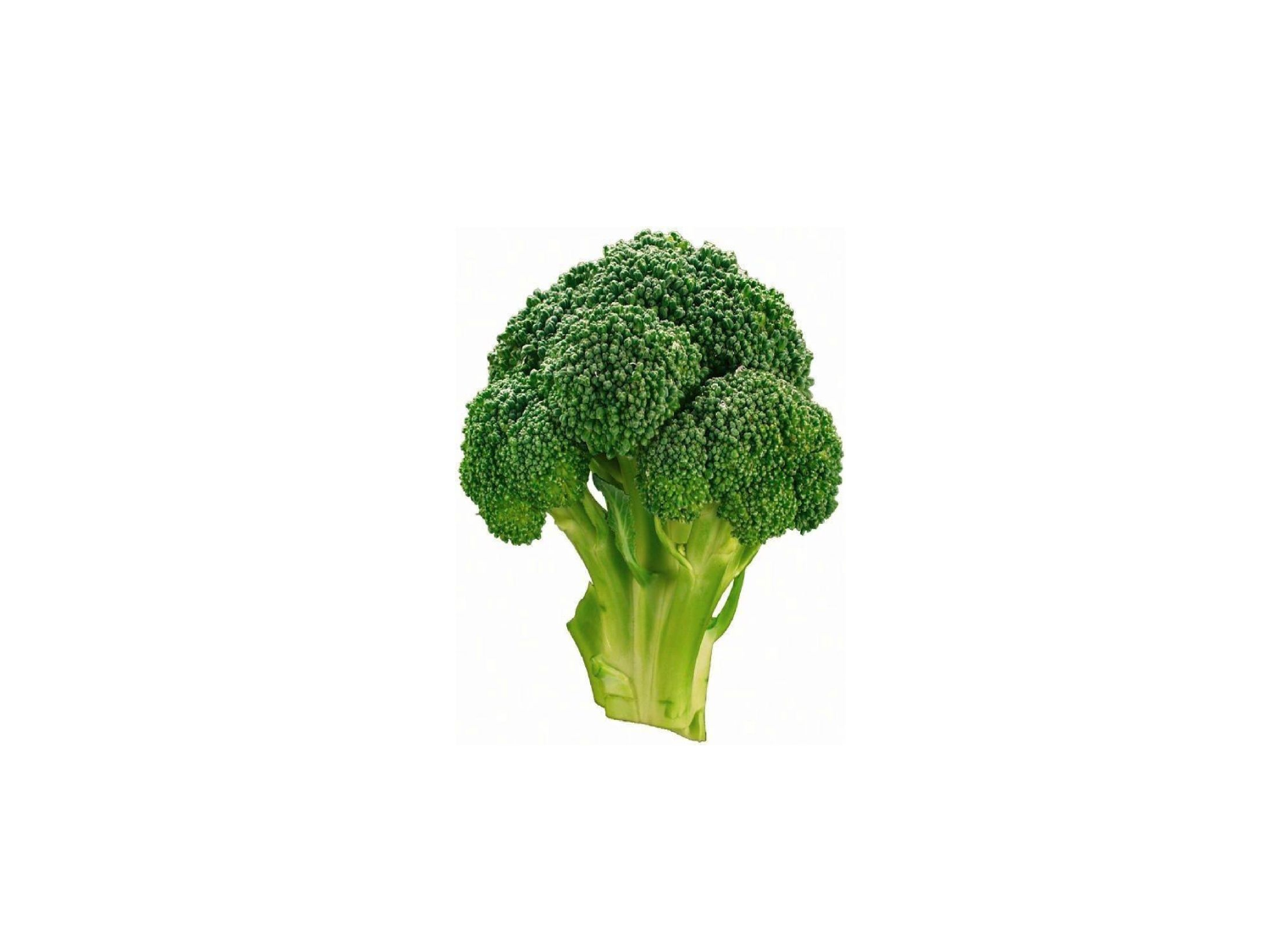

## Slide 20
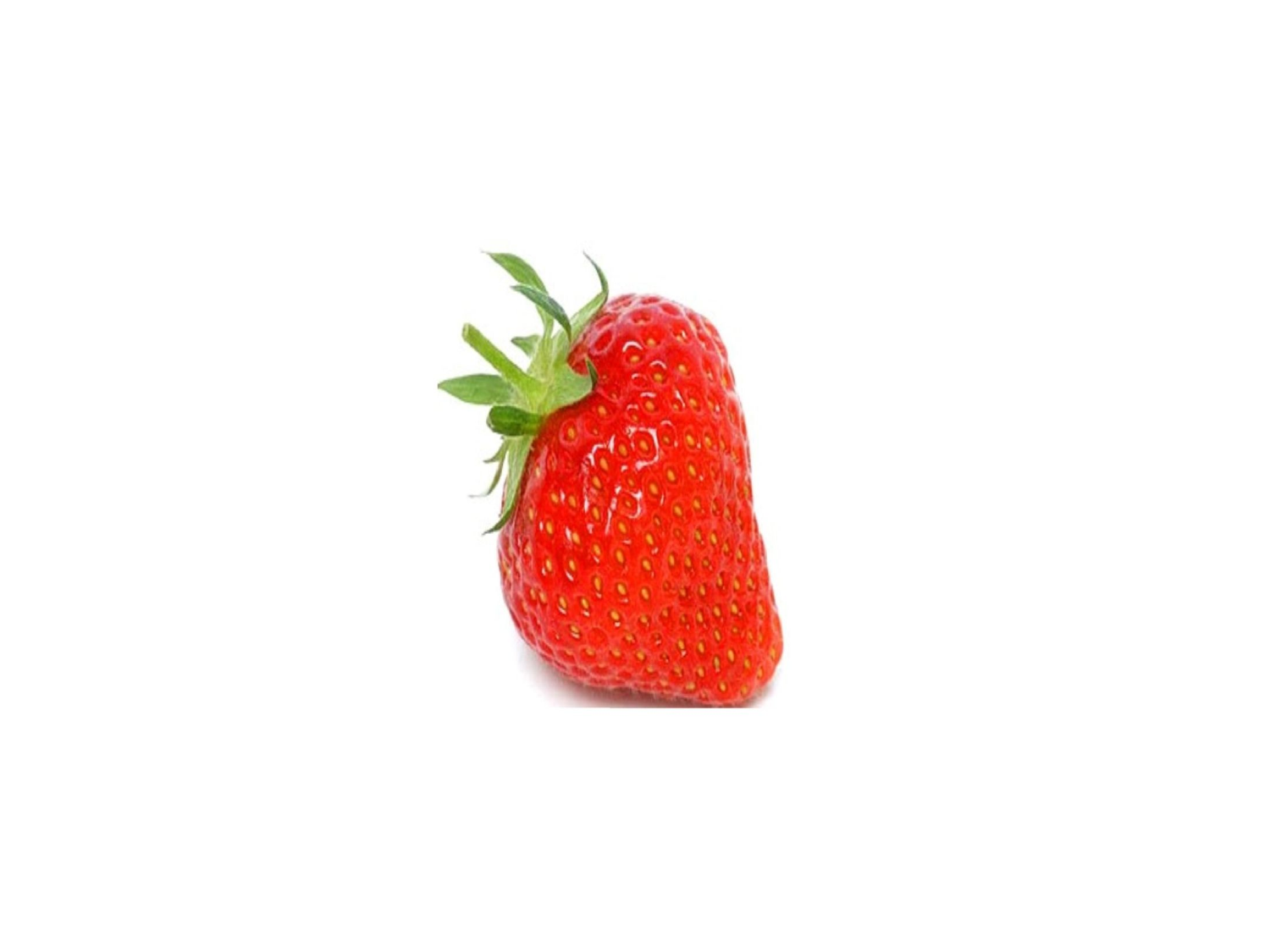

## Slide 21
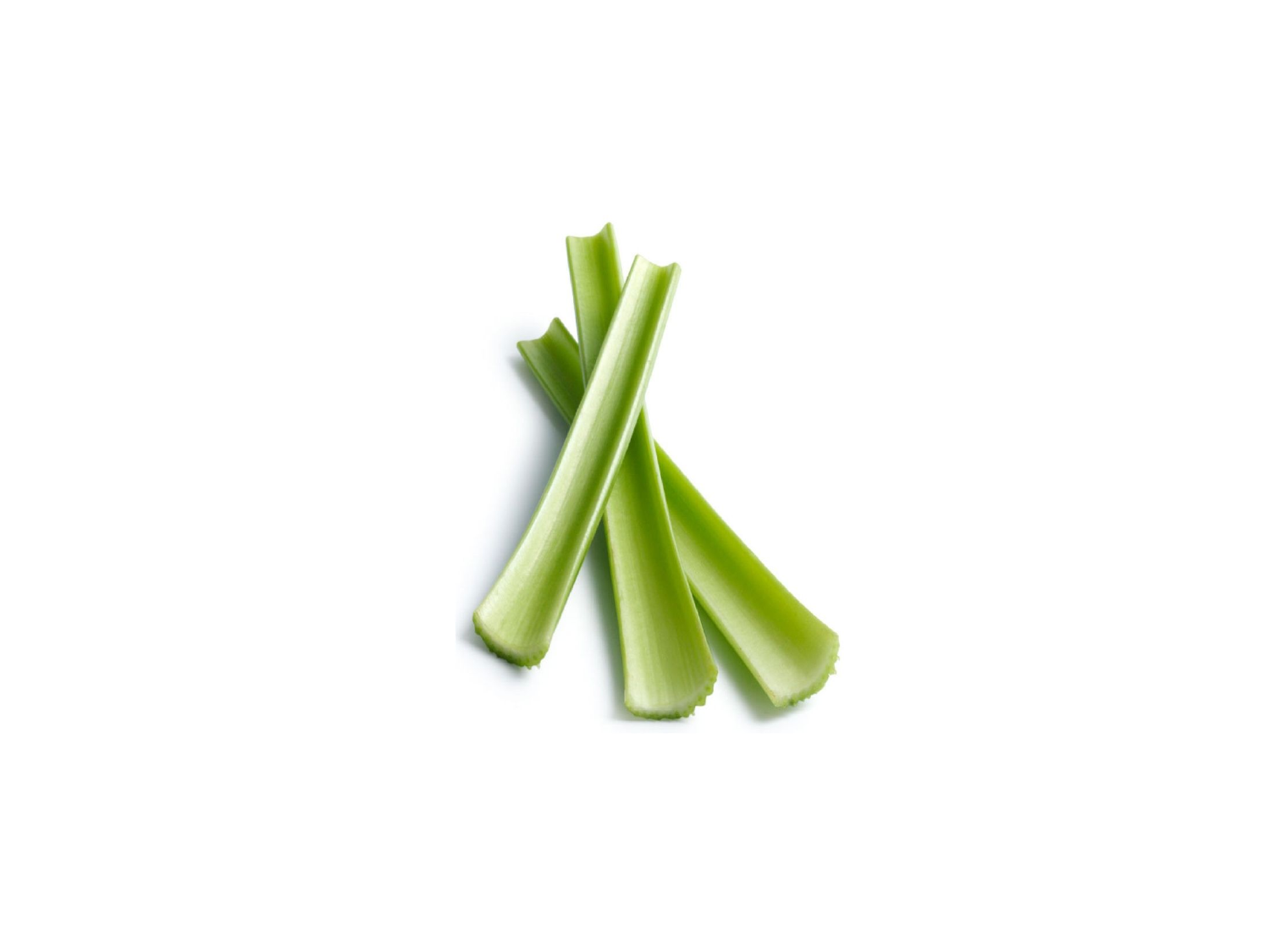

## Slide 22
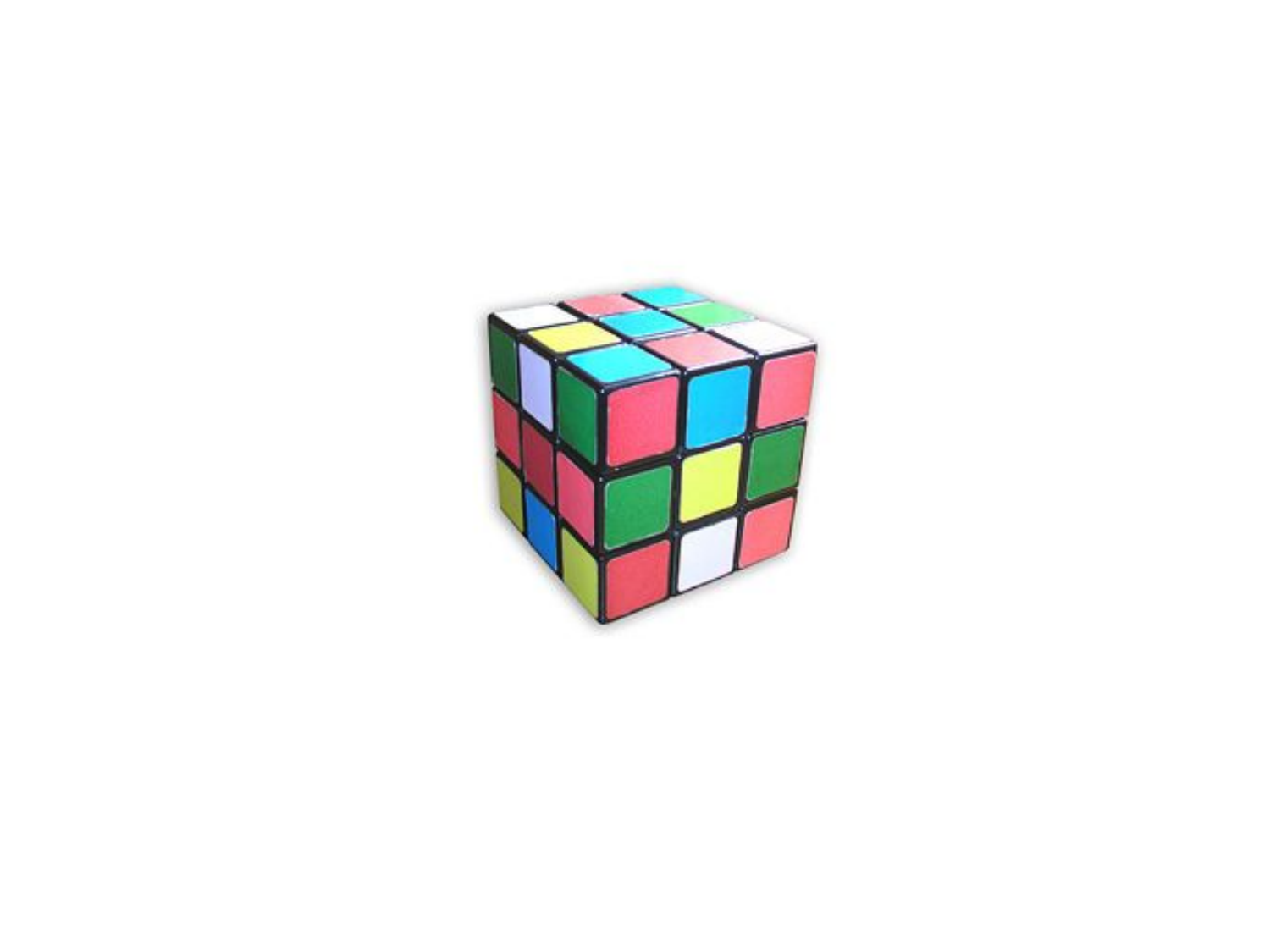

## Slide 23
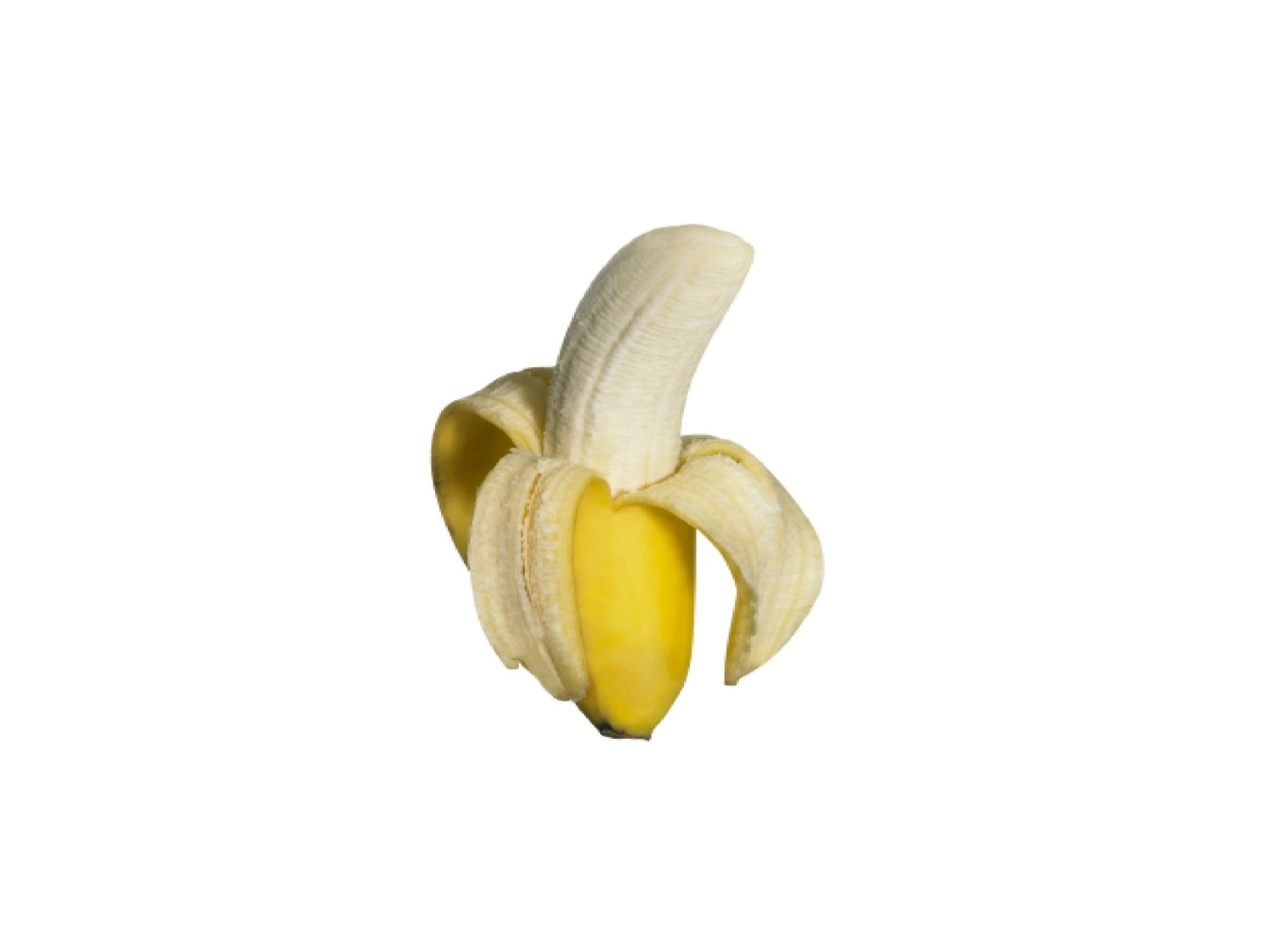

## Slide 24
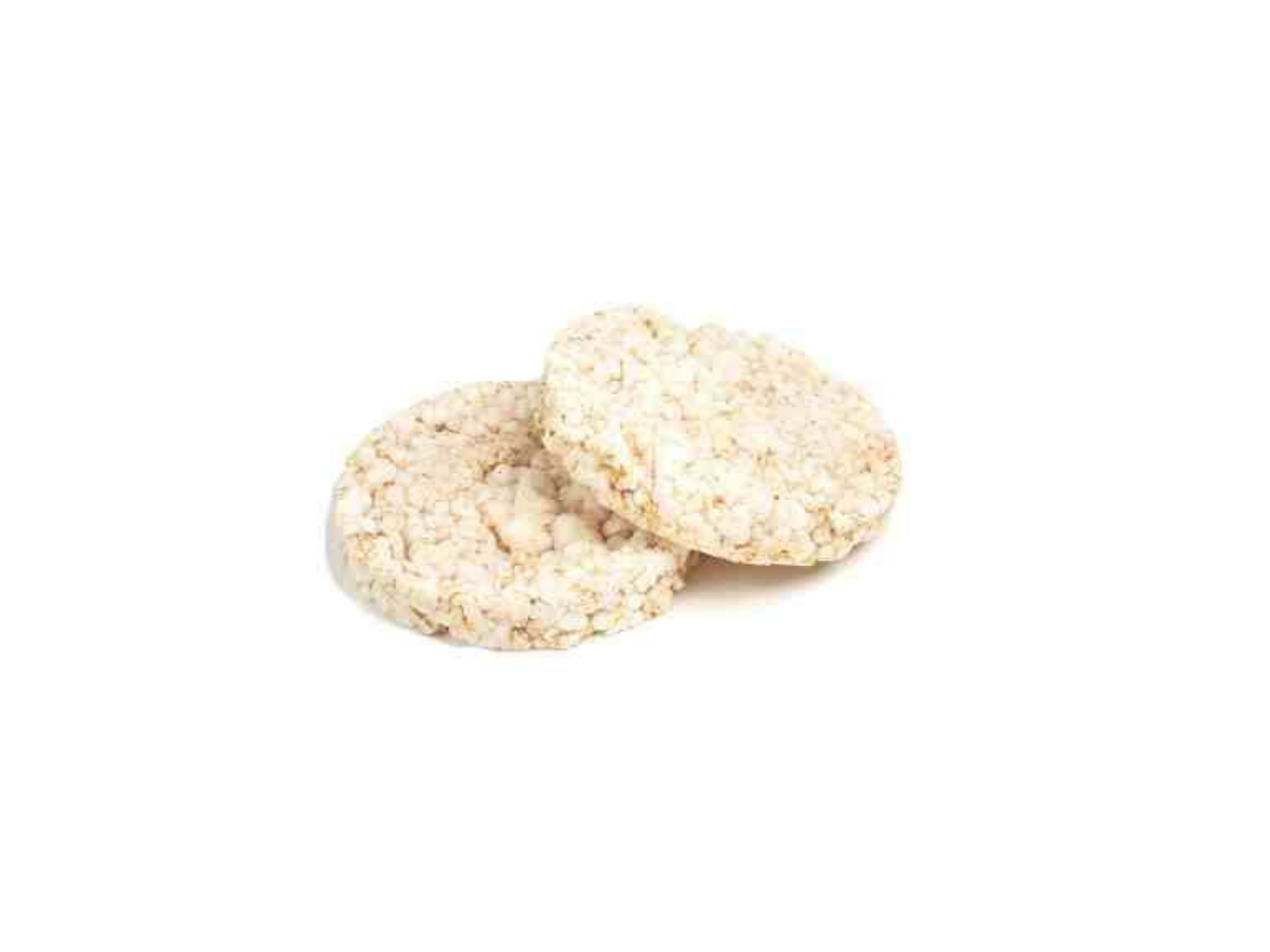

## Slide 25
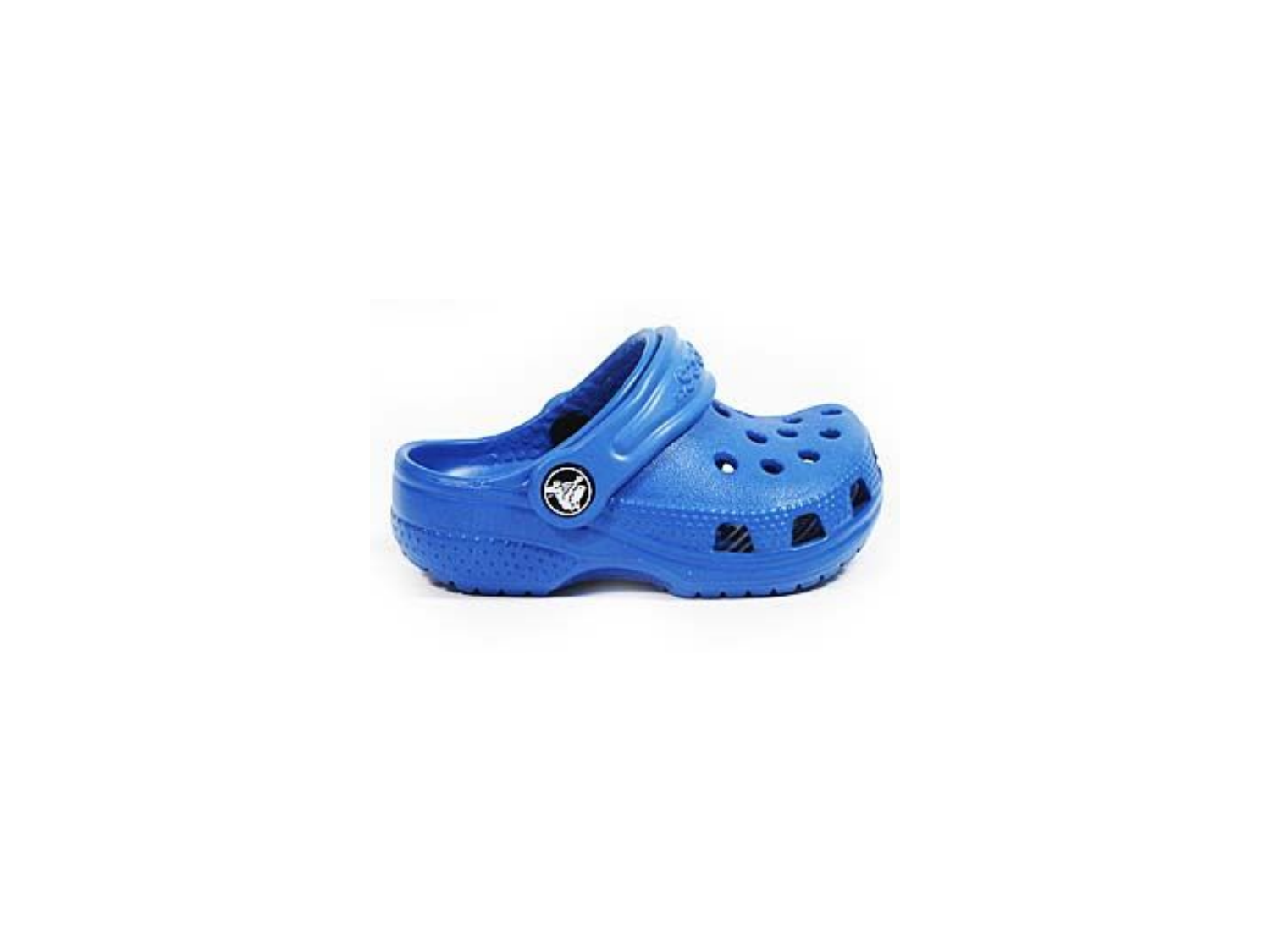

## Slide 26
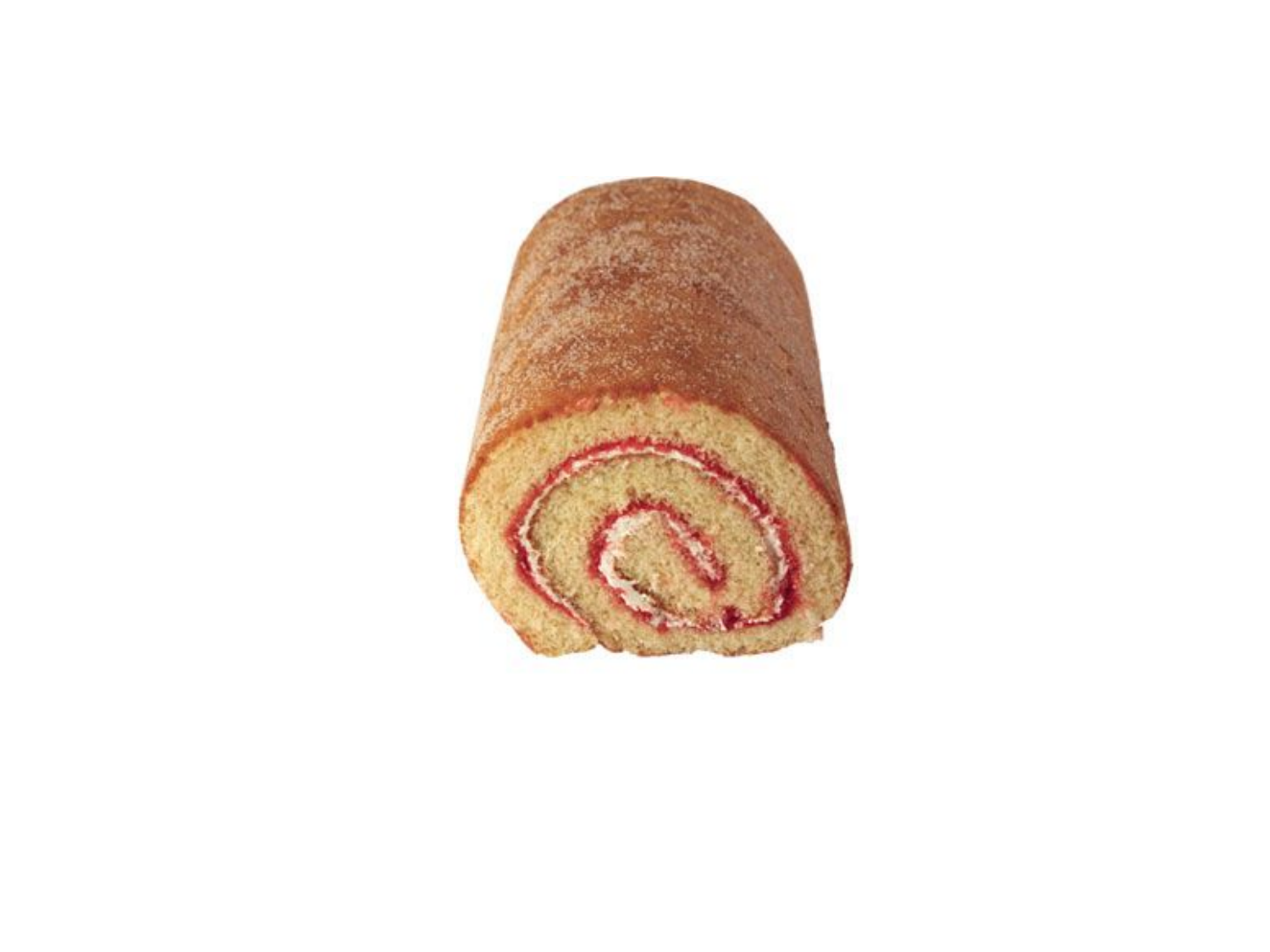

## Slide 27
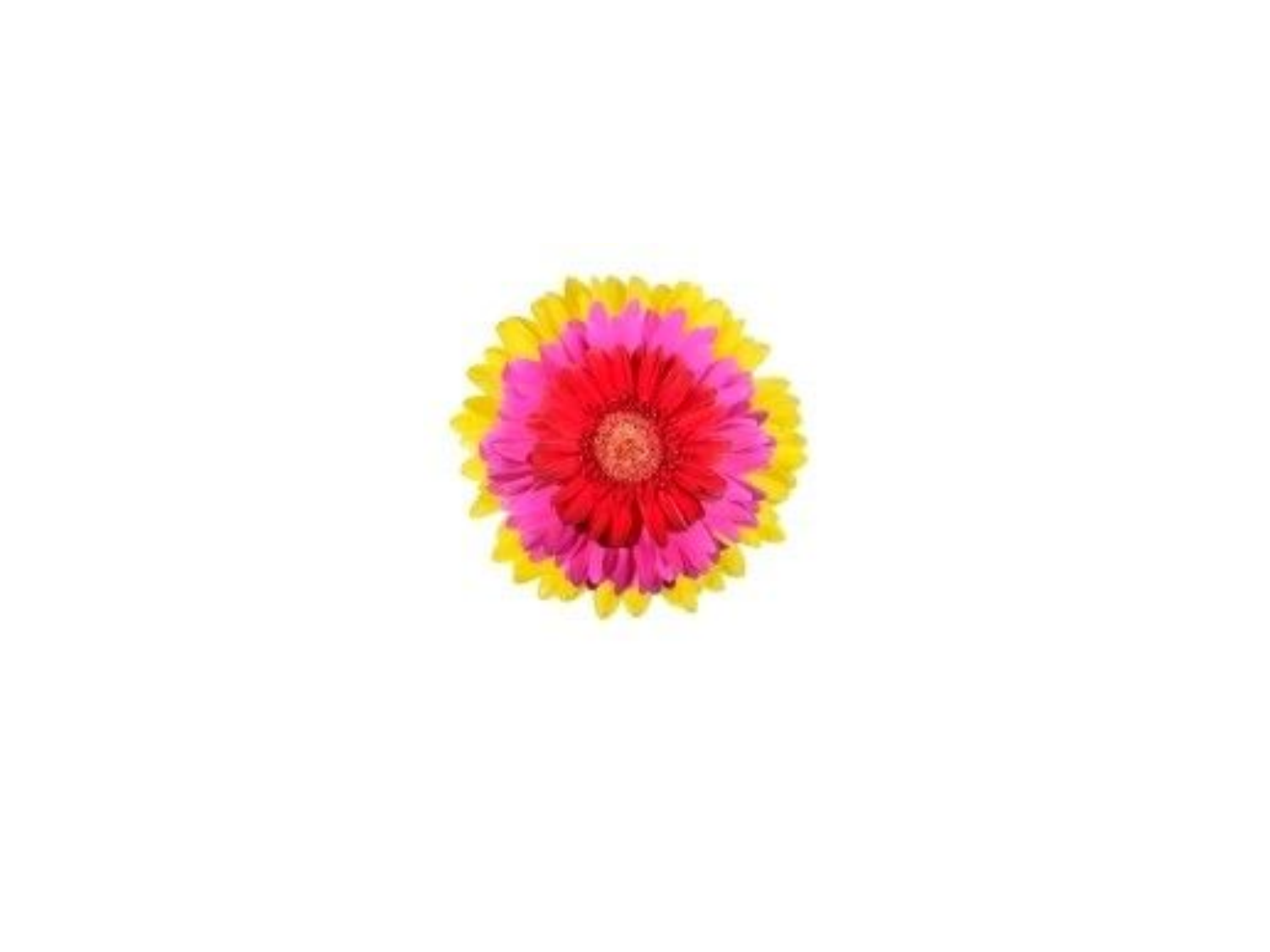

## Slide 28
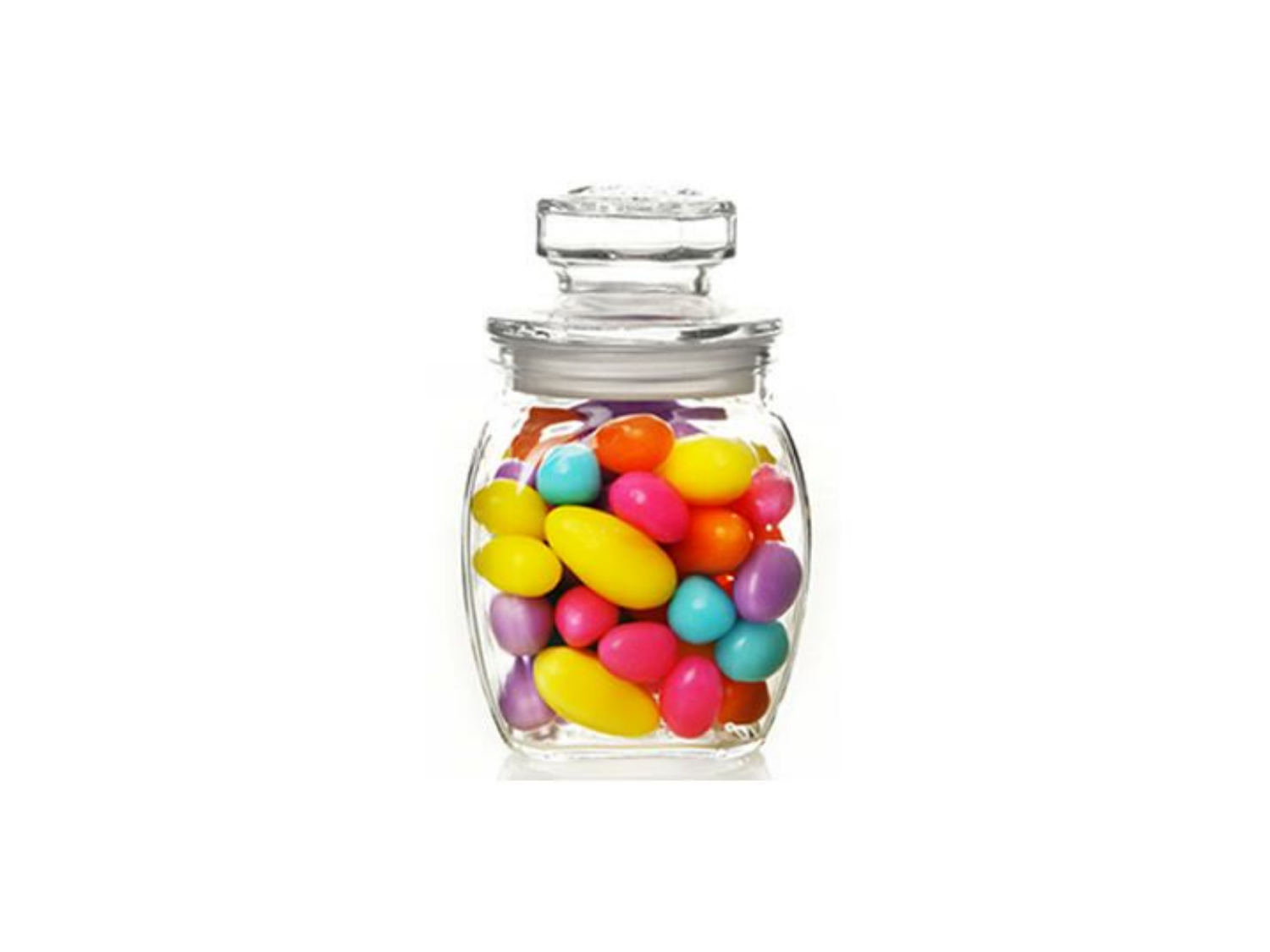

## Slide 29
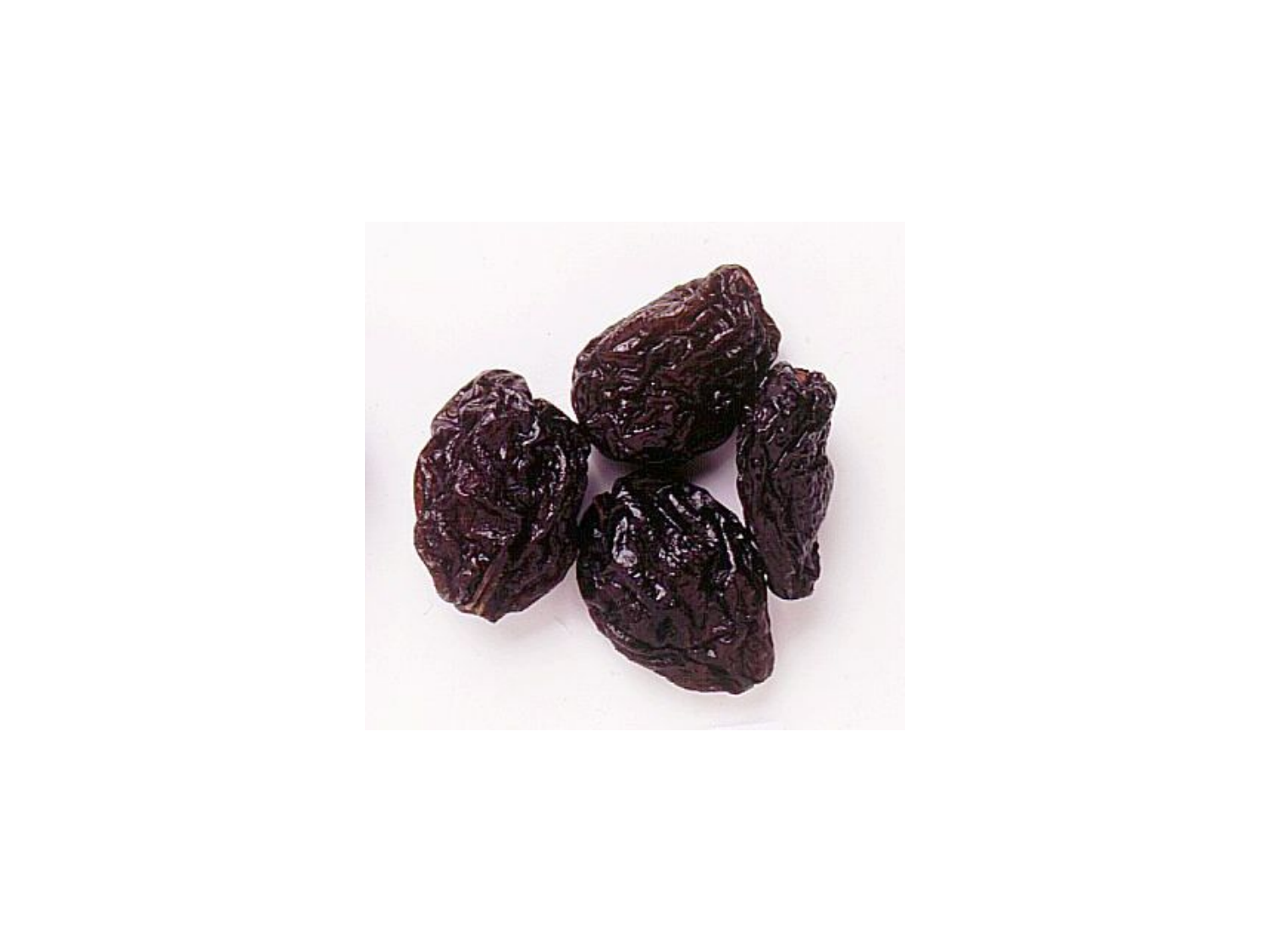

## Slide 30
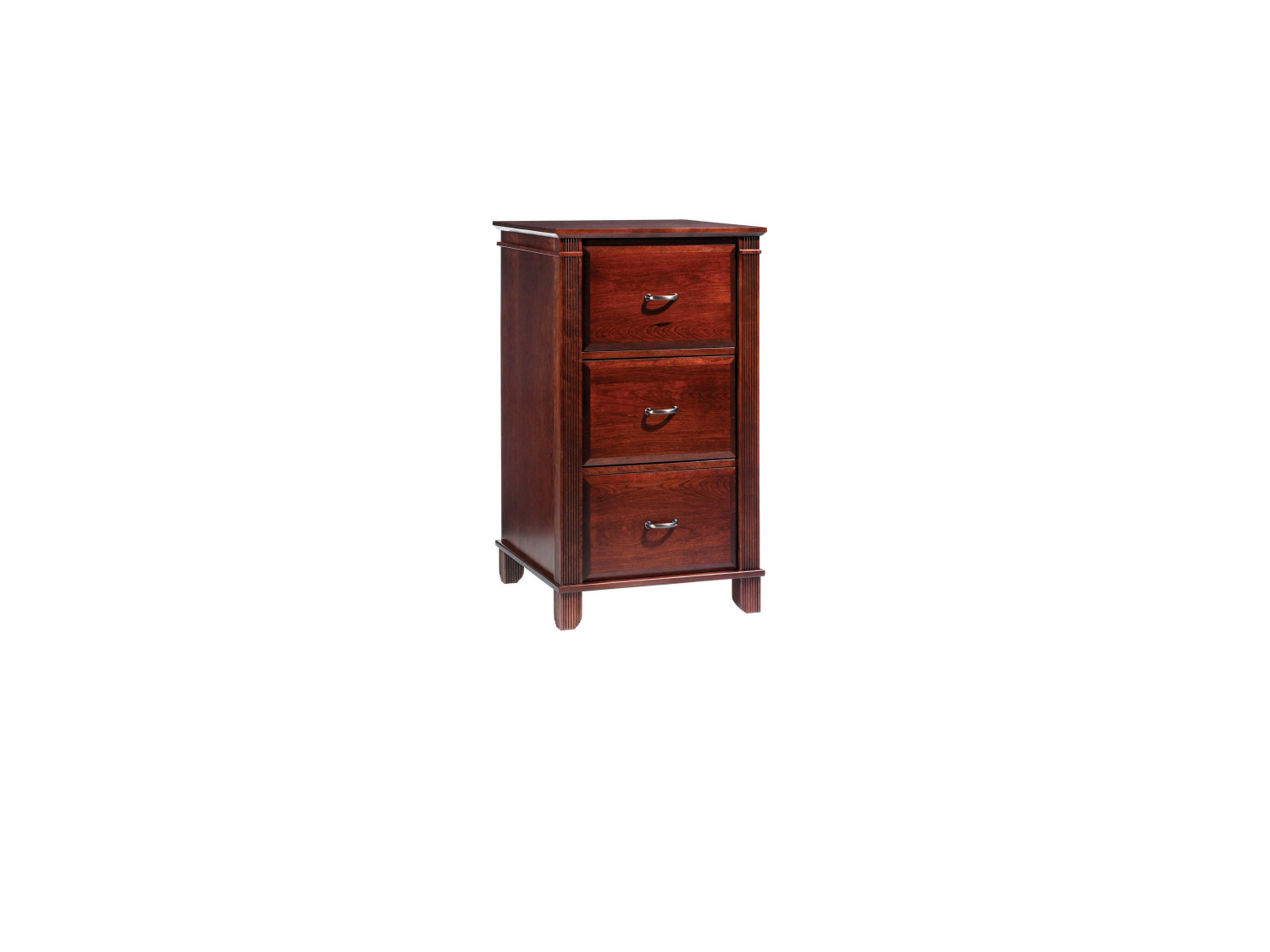

## Slide 31
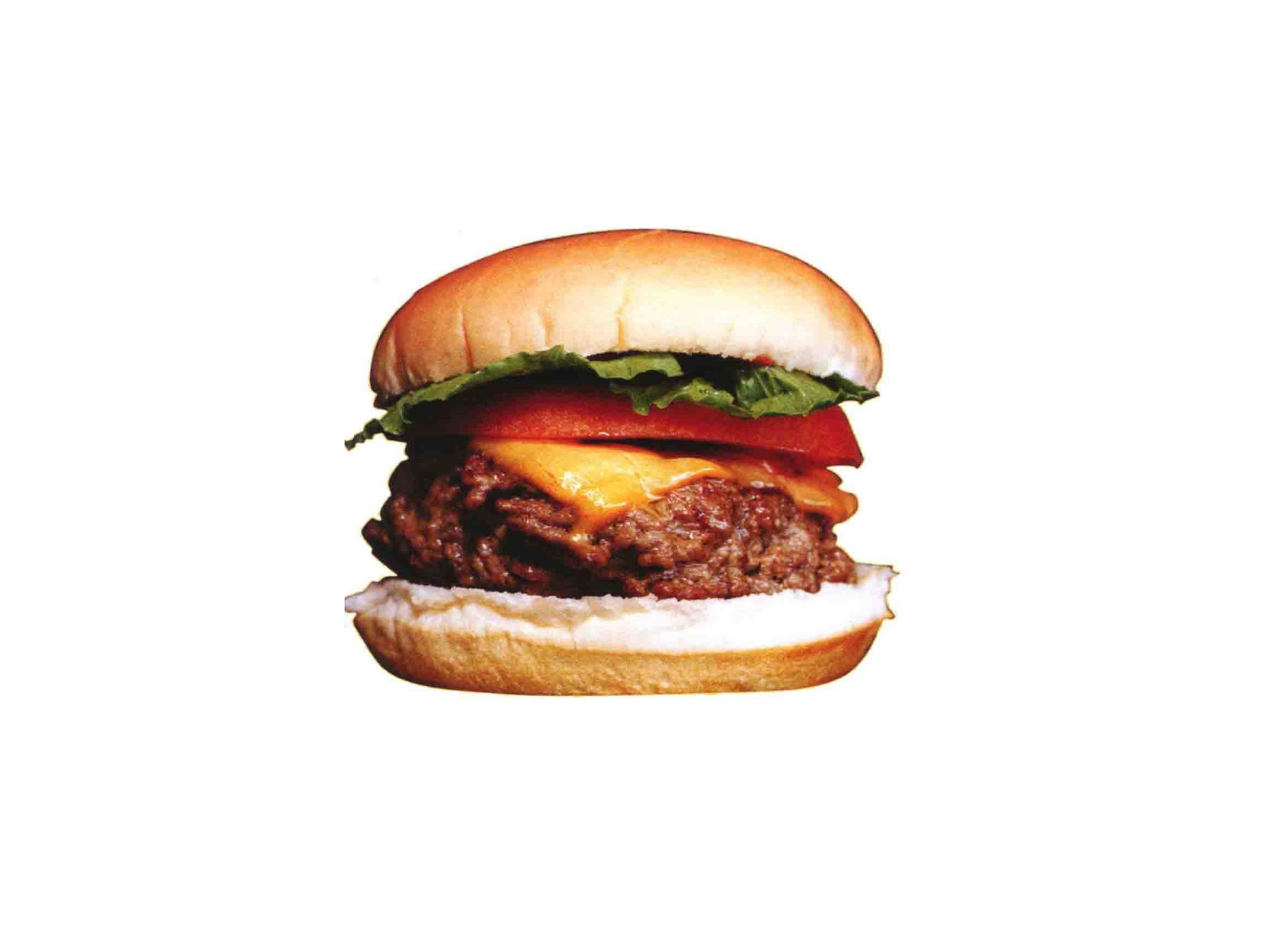

## Slide 32
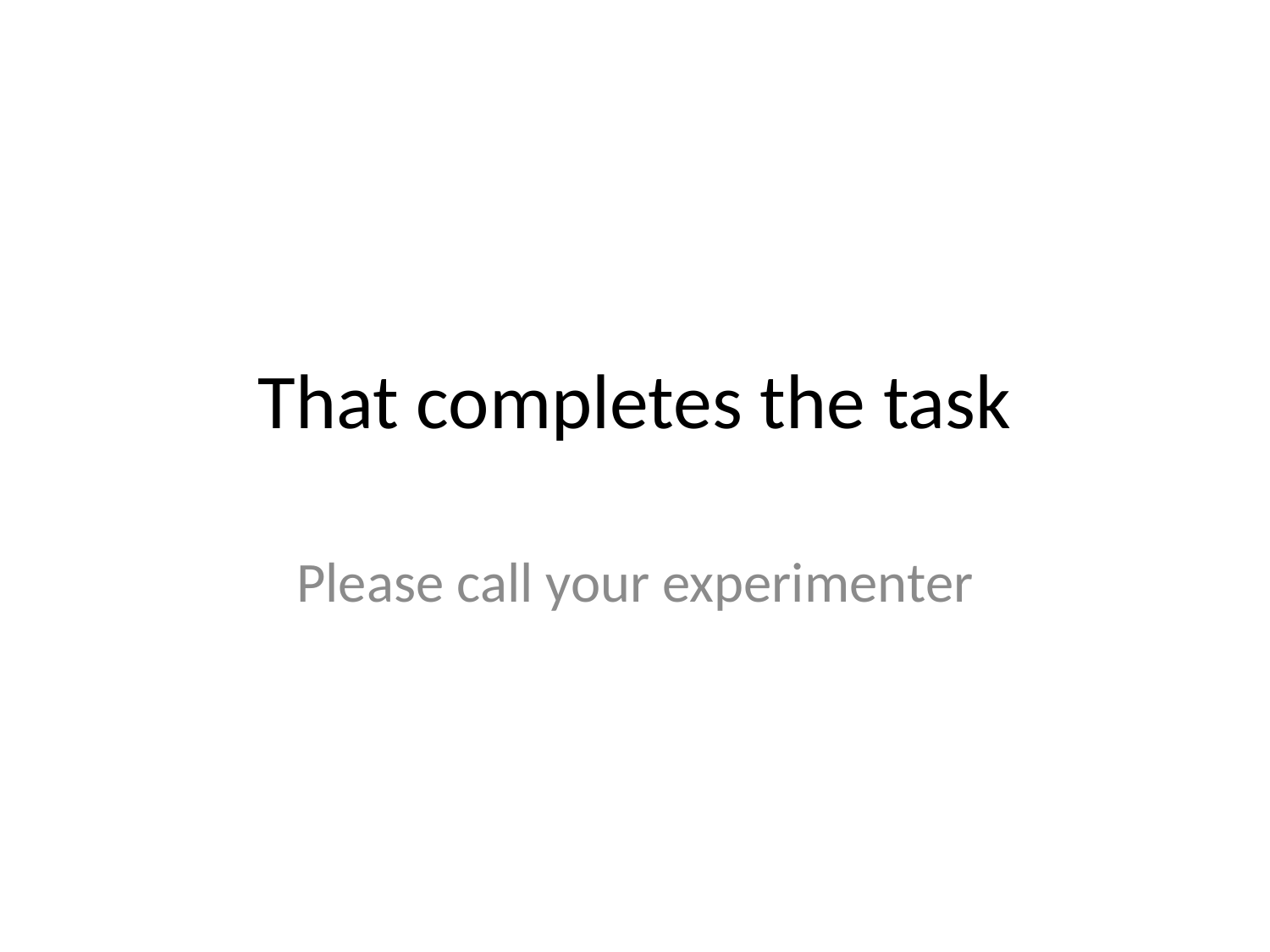

# That completes the task
Please call your experimenter
